# Leukemic mutation FLT3-ITD is retained in dendritic cells and disrupts their homeostasis leading to expanded Th17 frequency

**DOI:** 10.3389/fimmu.2024.1297338

**Published:** 2024-03-01

**Authors:** Patrick A. Flynn, Mark D. Long, Yoko Kosaka, Nicola Long, Jessica S. Mulkey, Jesse L. Coy, Anupriya Agarwal, Evan F. Lind

**Affiliations:** ^1^ Molecular Microbiology and Immunology, Oregon Health & Science University, Portland, OR, United States; ^2^ Department of Biostatistics and Bioinformatics, Roswell Park Comprehensive Cancer Center, Buffalo, NY, United States; ^3^ Department of Hematology & Medical Oncology, Knight Cancer Institute, Oregon Health & Science University, Portland, OR, United States; ^4^ Cell, Developmental and Cancer Biology, Oregon Health & Science University, Portland, OR, United States; ^5^ Division of Oncological Sciences, Oregon Health & Science University, Portland, OR, United States; ^6^ Knight Cancer Institute, Oregon Health & Science University, Portland, OR, United States

**Keywords:** dendritic cells, T helper (T) 17 cells, Treg, FLT3, AML, single cell

## Abstract

Dendritic cells (DC) are mediators between innate and adaptive immune responses to pathogens and tumors. DC development is determined by signaling through the receptor tyrosine kinase Fms-like tyrosine kinase 3 (FLT3) in bone marrow myeloid progenitors. Recently the naming conventions for DC phenotypes have been updated to distinguish between “Conventional” DCs (cDCs) and plasmacytoid DCs (pDCs). Activating mutations of FLT3, including Internal Tandem Duplication (FLT3-ITD), are associated with poor prognosis for acute myeloid leukemia (AML) patients. Having a shared myeloid lineage it can be difficult to distinguish *bone fide* DCs from AML tumor cells. To date, there is little information on the effects of FLT3-ITD in DC biology. To further elucidate this relationship we utilized CITE-seq technology in combination with flow cytometry and multiplex immunoassays to measure changes to DCs in human and mouse tissues. We examined the cDC phenotype and frequency in bone marrow aspirates from patients with AML to understand the changes to cDCs associated with FLT3-ITD. When compared to healthy donor (HD) we found that a subset of FLT3-ITD+ AML patient samples have overrepresented populations of cDCs and disrupted phenotypes. Using a mouse model of FLT3-ITD+ AML, we found that cDCs were increased in percentage and number compared to control wild-type (WT) mice. Single cell RNA-seq identified FLT3-ITD+ cDCs as skewed towards a cDC2 T-bet^-^ phenotype, previously shown to promote Th17 T cells. We assessed the phenotypes of CD4^+^ T cells in the AML mice and found significant enrichment of both Treg and Th17 CD4^+^ T cells in the bone marrow and spleen compartments. *Ex vivo* stimulation of CD4^+^ T cells also showed increased Th17 phenotype in AML mice. Moreover, co-culture of AML mouse-derived DCs and naïve OT-II cells preferentially skewed T cells into a Th17 phenotype. Together, our data suggests that FLT3-ITD+ leukemia-associated cDCs polarize CD4^+^ T cells into Th17 subsets, a population that has been shown to be negatively associated with survival in solid tumor contexts. This illustrates the complex tumor microenvironment of AML and highlights the need for further investigation into the effects of FLT3-ITD mutations on DC phenotypes and their downstream effects on Th polarization.

## Introduction

Dendritic cells (DCs) are cellular sentinels of the immune system that have evolved to detect pathogenic microorganisms, maintain tolerance to self, and prime T cells to their cognate antigens and contribute to protective immunity. In the decades after their formal description by Steinman and Cohn in 1979 (1, 2), the DC biology field has dramatically improved our understanding of both DC ontogeny and phenotype, which now includes cDC1, cDC2-subsets, and pDCs (3–8). Among these findings is the key developmental signal for cDCs during hematopoiesis through the engagement of fms-like tyrosine kinase 3 (FLT3) by its ligand FLT3L and downstream activation of STAT3 (9). Exogenous injection of FLT3L increases abundance of cDCs *in vivo* but there have not been reports of pathogenicity associated with high amounts of FLT3L (9–12).

Acute myeloid leukemia (AML) is a heterogeneous cancer of hematopoiesis that develops from the combination of two or more classes of mutations that affect proliferation, differentiation, and epigenetics of myeloid precursors in the bone marrow (13). Activating mutations of FLT3 have a strong association with leukemias including AML (13–15). It is the most common mutation found in AML patients, up to 30%, acquired by internal tandem duplications (FLT3-ITD) of the cytoplasmic domain of FLT3 (14, 16–20) and causes ligand independent signaling (16, 21). There are few reports characterizing blood DC-subsets in both human leukemia and myelodysplastic syndromes (22, 23) and sample sizes are limited. Recently it has been reported that some AML tumor cells may express CD11c, a key marker of DC identification *in vivo* (24). Because of the genetic variability of the disease, it has been difficult to distinguish *bona fide* DCs from tumor cells with aberrant expression of DC-related proteins.

Mouse models to study AML have diverse mutation targets and cover both inducible and constitutive expression models with varying degrees of disease induction (25). Despite the variety of models available, there is a paucity of DC-specific reports. It was shown in a non-leukemia model that the FLT3-ITD mutation produces functional DCs without pathogenic side effects but does result in increased DC abundance and moderate effects on CD4^+^ T cell phenotype (26).

To provide insight into the phenotypic and molecular changes in DCs during AML we investigated samples isolated from patients with AML and a genetically-engineered mouse model (GEMM) of AML that spontaneously develops disease. We first identified a disruption in the frequency of cDCs in the bone marrow of patients with AML segregated by their FLT3-ITD status, suggesting that some AML patients have increased output of DCs compared to healthy donors. We then utilized our GEMM AML mice and confirmed that the FLT3-ITD mutation causes significant expansion of cDCs both in the bone marrow and spleens of AML mice compared to FLT3-ITD**^-^** controls and healthy mice. Based on that finding we used single cell RNA-sequencing (scRNA-seq) to interrogate changes to AML cDCs at the transcript level and correlated the expression of surface proteins using antibody-derived tags (ADTs). We were able to identify *bona fide* cDCs transcriptionally and found an enrichment of the recently described T-bet**^-^** cDC2 that are efficient at polarizing naïve CD4^+^ T cells into Th17 cells. In the mice with AML, we observed elevated levels of blood serum cytokines that are permissive to Th17 polarization of CD4^+^ T cells. There is a significant enrichment of both Treg and Th17 phenotypes in the blood of AML mice, suggesting that AML DCs may be driving their polarization. To measure the impact of DCs on T cell skewing we used adoptive transfer studies to compare AML and WT healthy mice. We did not find Treg or Th17 polarization; however, AML hosts expanded and retained significantly more transferred OT-II cells compared to WT, suggesting that the increased DC frequency supports naïve OT-II T cell survival. Finally, we show that co-culturing naïve OT-II cells with AML DCs results in increased IL-17A production, suggesting that AML DCs play a role in polarizing Th17 cells in the presence of cognate antigen. Together, we propose that DC-progenitors retain the FLT3-ITD mutation which drives their significant expansion and leads to the activation of naïve CD4^+^ T cells that polarize into Th17 and Treg phenotypes. These T cell subsets would likely result in negative impacts to anti-tumor responses in AML.

## Materials and methods

### Human samples

Flow cytometry analisis was performed on 22 samples, 12 FLT3 WT and 10 FLT3-ITD+. Sample characteristics including mutational status, surface phenotype and karyotype are displayed in **Supplementary Table 1**. Bone marrow aspirates were separated by Ficoll density gradient centrifugation and frozen using a cocktail of 90% FBS+10% DMSO for cryogenic storage in liquid nitrogen. All human flow cytometry experiments were performed using liquid nitrogen stored samples. Samples were carefully thawed using a 37°C water bath before immediate slow transfer into warm 10% FBS 1X DMEM media and centrifugated at 300xg for 10 minutes. DNAse-I was added to pelleted cells for a final concentration of 50 µg/mL and 10 mL of warm media to resuspend cell pellet. Any bone fragments remaining were filtered using 70 µm mesh filters before antibody staining. Viability was determined by Zombie Aqua staining and doublets were gated out of analysis by FSC-A vs. FSC-H. Flow cytometry data were acquired on a BD LSRFortessa or Cytek Aurora and analyzed using FlowJo v10 software. All human sample experiments are approved under IRB protocol #00004422, “Pathogenesis of Acute Leukemia, Lymphoproliferative Disorder and Myeloproliferative Disorders” (PI: Marc Loriaux, MD, PhD). Informed consent was obtained from all patients. Human antibody clones and sources are listed in **Table 1**.

**Table 1:** Human antibody list.

Antigen	Fluor	Clone	Catalogue	Vendor
AXL	Alexa Fluor 350	108724	FAB154U-100G	BD Bio
XCR1	BV421	S15046E	372610	BioLegend
CD11b	Pacific Blue	ICRF44	558123	BD Bio
Zombie Aqua			423102	BioLegend
CD45	BV605	HI30	304042	BioLegend
CD123	BV650	6H6	306020	BioLegend
CD33	BV711	WM53	303424	BioLegend
CD19	BV786	HIB19	302240	BioLegend
CD11c	Alexa Fluor 488	Bu15	337236	BioLegend
CD3e	PerCP	OKT3	317338	BioLegend
CD34	PerCP-Cy5.5	561	343612	BioLegend
CLEC10A	PE	H037G3	354704	BioLegend
CD56	PE Dazzle 594	HCD56	318348	BioLegend
HLA-DR	PE-Cy5	L243	307608	BioLegend
CD1c	PE-Cy7	L161	331516	BioLegend
CD38	Alexa Fluor 647	HH2	303514	BioLegend
CD14	Alexa Fluor 700	HCD14	325614	BioLegend

### AML murine model

Mice expressing FLT3-ITD under the endogenous FLT3 promoter (strain B6.129-Flt3tm1Dgg/J, The Jackson Laboratory, stock no. 011112) (27) were crossed to mice with the TET2 gene flanked by LoxP sites (strain B6;129STet2tm1.1Iaai/J, The Jackson Laboratory, stock no. 017573) (28). The Flt3ITD/Tet2 flox mice were then crossed to mice expressing Cre recombinase under the Lysm promoter (strain B6.129P2-Lyz2tm1(cre)Ifo/J, The Jackson Laboratory, stock no. 004781). The Flt3ITD/Tet2/LysMCre mice were bred to mice with the TP53 gene flanked by LoxP sites (strain B6.129P2-Trp53tm1Brn/J, The Jackson Laboratory, stock no. 008462). All breeding animals were purchased from The Jackson Laboratory. All mice used in these experiments were bred as heterozygous for FLT3-ITD and LysCre but homozygous for TET2 and TP53. All mouse experiments were performed in accordance with the OHSU Institutional Animal Care and Use Committee protocol IP00000907. No inclusion or exclusion criteria were used on the animals with correct genotype. Mice were selected and assigned to groups randomly while maintaining a 50% male 50% female ratio per experiment. No blinding was performed. Average age of mice used for *in vivo* studies was 30 weeks.

### *Ex vivo* cell preparation of mouse splenocytes

Spleens were harvested from mice and mechanically dissociated using frosted microscope slides then rinsed with 1X PBS. Single cell suspensions were passed through 70-µm cell strainers and red blood cells were then lysed with ammonium chloride-potassium (ACK) lysis buffer. Cells were counted with hemacytometer and 3–5 × 10^6^ cells were used per antibody-staining reaction. For experiments requiring enrichment of splenic cDCs, cells were enriched from total spleen cells with MojoSort™ Mouse CD11c Nanobeads (BioLegend Cat: 480078) according to manufacturer’s protocol. After positive selection cells were counted via hemacytometer to assess viability and cell count with purity assessed via flow cytometry. For adoptive transfer and co-culture experiments naïve OT-II cells were magnetically enriched with MojoSort™ Mouse CD4 Naïve T Cell Isolation Kit (BioLegend Cat: 480040) according to manufacturer’s protocol. After positive selection cells were counted via hemacytometer to assess viability and cell count with purity assessed via flow cytometry.

### Flow cytometry staining of mouse tissues

Bone marrow, blood, or splenocytes were processed and subjected to red blood cell lysis by ACK before counting via hemacytometer. Cells were resuspended in 1X PBS and stained at 4°C with 100 µL 1:500 Zombie Aqua viability dye (BioLegend, Cat# 423102) and 1:200 mouse FC block (TruStain FcX, BioLegend Cat# 101320) for 15 min, covered from light. Cells were pelleted for 5 minutes at 300xg. 100 µL of cell surface staining antibody cocktail was added directly on top of the cells for resuspension and stained on ice for 30 min in 1X FACS buffer (1X PBS, 1.5% calf serum, 0.02% sodium azide, 2mM EDTA). For intracellular staining, the cells were then washed with FACS buffer, permeabilized, and stained for intracellular targets according to the manufacturer’s protocol (eBioscience FOXP3 Transcription Factor Staining Buffer Set, Cat# 00-5523-00), then resuspended in 1X FACS buffer before analyzing on either a BD Fortessa or Cytek Aurora flow cytometer. Data were analyzed using FlowJo version 10 software. Mouse antibody clones and sources are listed in **Table 2**.

**Table 2:** Mouse antibody list.

Antigen	Fluor	Clone	Catalogue	Vendor
CD45	BUV496	30-F11	749889	BD Bio
CD86	BUV737	GL1	741737	BD Bio
1/A-I/E	BV421	M5/114.15.2	107631	BioLegend
CD80	BV650	16-10A1	104732	BioLegend
CD317/BST2/PDCA-1	BV711	927	104732	BioLegend
XCR1	BV785	ZET	148225	BioLegend
CD103	Alexa Fluor 488	2 E7	121408	BioLegend
CD135	PE	A2F10	135306	BioLegend
CD11b	PE-Cy7	M1/70	101216	BioLegend
F4/80	APC	BM8	123116	BioLegend
CD25	PE-Cy7	PC61	102026	BioLegend
CD45.1	BV711	A20	110739	BioLegend
CD11c	Alexa Fluor 488	N418	117311	BioLegend
Ly6G	PerCP-Cy5.5	1A8	127616	BioLegend
CD3e	PE	145-2C11	100308	BioLegend
SIRPa	PE-Dazzle 594	P84	144016	BioLegend
CD19	PE-Dazzle 594	605	115554	BioLegend
GR-1	BV605	RB6-8C5	108439	BioLegend
CD3e	BV421	145-2C11	100336	BioLegend
CD317/BST2/PDCA-1	BV711	927	127039	BioLegend
CD11b	APC	M1/70	101212	BioLegend
Ly6C	Alexa Fluor 700	HK1.4	128024	BioLegend
CD621	APC-Cy7	MHL-14	104428	BioLegend
CD86	PE-Cy7	PO3	105116	BioLegend
CD86	BV650	GL-1	105035	BioLegend
XCR1	BV650	ZET	148220	BioLegend
CD86	APC-Cy7	GL-1	105030	BioLegend
B220	PerCP-Cy5.5	RA3-682	103236	BioLegend
1/A-I/E	PE	M5/114.15.2	107608	BioLegend
IFNy	PacificBlue	XMG1.2	505818	BioLegend
IL-4	BV605	11B11	504126	BioLegend
1/A-I/E	BV650	M5/114.15.2	107641	BioLegend
IL-17A	BV711	TCH11-18H10.1	506941	BioLegend
FOXP3	PE-CY7	FJK.16S	25-5773-82	eBioscience
GATA3	APC	16E10A23	653805	BioLegend
T-bet	BV786	04-46	564141	BioLegend
RORyt	PE	B2D	12-6981-82	eBioscience
CD25	APC-R700	PC61	565134	BD Bio
CD45.2	APC-Cy7	104	109824	BioLegend
CD11c	BV421	N418	117330	BioLegend
CD8a	PacificBlue	53-6.7	100725	BioLegend
CD44	BV605	IM7	103407	BioLegend
NK1.1	BV650	PK136	108736	BioLegend
CD19	BV711	605	115555	BioLegend
CD3e	Alexa Fluor488	145-2C11	100321	BioLegend
I/A-I/E	PerCP	M5/114.15.2	107624	BioLegend
CD4	PerCP-Cy5.5	RM4-4	116012	BioLegend
Zombie Aqua			423102	BioLegend

### Adoptive transfer experiments

Spleens from CD45.2^+^ OT-II mice (29) were harvested from mice and prepared for naïve OT-II cell transfer as described above before transfer to AML CD45.1^+^ or WT CD45.1^+^ recipient mice. On Day 0 200,000 naïve OT-II cells were intravenously injected into CD45.1^+^ recipient mice. On day 1 soluble whole-OVA protein (200 µg) or control whole-BSA protein (200 µg) was injected into recipient mice. On Day 11 spleens from recipient mice were harvested and splenocytes were processed for flow cytometry staining to assess OT-II populations for cell number and frequency as described above.

### *Ex vivo* stimulation of splenocytes for cytokine secretion

Splenocytes from WT and AML mice were processed and subjected to red blood cell lysis by ACK before counting via hemacytometer. 500,000 cells were placed into tissue-culture treated 96-well plates that were treated with anti-mouse CD3ϵ IgG antibody (BioLegend Cat: 100340; Clone: 145-2C11) at a final concentration of 2.5 µg/mL plus anti-mouse CD28 (BioXcell Cat: BE0015-1; Clone: 37.51) at a final concentration of 20 µg/mL or control Armenian Hamster IgG antibody (BioLeged Cat: 400959; Clone: HTK888) at a final concentration of 2.5 µg/mL for Unstimulated control group and 1X Cell Activation Cocktail with Brefeldin A (CAC) (BioLegend Cat: 423303). Cells were then incubated for 6-hours in 1X Culture Media (10% FBS, 1% Pen/Strep, 1X MEM, 1 µM HEPES, 50 µM 2-ME). Cells were harvested after incubation, washed 2x with 1X PBS before processing for intracellular flow cytometry.

### LegendPlex blood serum assay

Peripheral blood from WT and AML was collected via retro-orbital sources. Blood was collected in Sarstedt Microvette^®^ 500 Serum Gel tubes and centrifugated at 10,000 x G for 5 minutes at room temperature to remove cells from the serum. Aliquots of the remaining serum were stored at -80°C before assayed. On day of being assayed aliquots were thawed slowly on ice before being tested using the flow cytometry based LEGENDplex™ Mouse Inflammation Panel (BioLegend Cat: 740446) according to the manufacturer’s instructions for sample processing. Samples were measured on a BD Fortessa according to the manufacturer’s instructions for calibration and data collection. Data was analyzed using the online resource from BioLegend (https://legendplex.qognit.com).

### ADTs and Single cell RNA-seq

Spleens from WT and AML mice were harvested and processed into filtered single cell suspensions as described above. After counting, cells were enriched for DC populations using negative selection EasySep™ Mouse Pan-DC Enrichment Kit II (StemCell Cat: 19863) according to the manufacturer’s protocol. After magnetic enrichment unlabeled cells were counted and aliquoted into 1x10^6^ total cells for TotalSeq™-A (BioLegend, custom panel) ADT staining. Cells were incubated with 1:200 TruStain FcX™ PLUS (BioLegend Cat: 156604) in a final volume of 50 µL 1X FACS buffer for 10 minutes at 4°C. ADT 2X cocktail master mix was prepared for a final dilution of approximately 1 µg per ADT as follows: 1.8 µL per ADT for a total volume of 37.8 µL of just ADTs. 412.2 µL of 1X FACS buffer added to the ADTs for a final volume of 450 µL 2X ADT master mix cocktail. Add 50 µL of the master mix to each sample for a final volume of 100 µL. Incubate samples for 30 minutes at 4°C. Add 100 µL 1X FACS buffer and pellet cells for 5 minutes at 300xg. Wash two more times to remove any unbound ADTs. Resuspend cell pellets at 1,000 cells per µL with 1X PBS 0.04% BSA. Samples were transferred to the OHSU Massively Parallel Sequencing Shared Resource (MPSSR) for 10X Genomics Chromium CITE-seq library and scRNA-seq cDNA library preparation according to 10X Genomics protocols. Each sample was sequenced on its own lane on the chip with no multiplexing. Each sample was sequenced with a target number of 10,000 cells per sample at a reads of 20,000 per cell for an approximate read depth of 200 million reads. List of ADTs and source are listed in **Table 3**.

**Table 3:** ADT list.

Antigen	Clone	Catalogue	Vendor
TotalSeq™-A0002 anti-mouse CD8a	53-6.7	100773	BioLegend
TotalSeq™-A0103 anti-mouse/human CD45R/B220 Antibody	RA3-682	103263	BioLegend
TotalSeq™-A0106 anti-mouseCD11cAntibody	N418	117355	BioLegend
TotalSeq™-A0014 anti-mouse/human CD11b Antibody	M1/70	101265	BioLegend
TotalSeq™-A0117 anti-mouse	M5/114.15.2	107653	BioLegend
TotalSeq™-A0200 anti-mouse CD86 Antibody	GL-1	105047	BioLegend
TotalSeq™-A0201 anti-mouse CD103 Antibody	2E 7	121437	BioLegend
TotalSeq™-A0212 anti-mouse CD24 Antibody	M1/69	101841	BioLegend
TotalSeq™-A0422 anti-mouse CD172a (SIRPa) Antibody	P84	144033	BioLegend
TotalSeq™-A0563 anti-mouse Q33R1 Antibody	SA011F11	149041	BioLegend
TotalSeq™-A0568 anti-mouse/rat XCR1 Antibody	ZET	148227	BioLegend
TotalSeq™-A0811 anti-mouse CD317 (BST2, PDCA-1) Antibody	927	127027	BioLegend
TotalSeq™-A0849 anti-mouse CD80 Antibody	16-10A1	104745	BioLegend
TotalSeq™-A0903 anti-mouse CD40 Antibody	3/23	124633	BioLegend
TotalSeq™-A1010 anti-mouse CD205 (DEC-205) Antibody	NIDC-145	138221	BioLegend
TotalSeq™-A0093 anti-mouse CD19 Antibody Antibody	6D5	115559	BioLegend
TotalSeq™-00981 anti-mouse TCR Va2 Antibody	B20.1	127831	BioLegend
TotalSeq™-00013 anti-mouse Ly-6C Antibody	HK1.4	128047	BioLegend
TotalSeq™-A0015 anti-mouse Ly-6G Antibody	1A8	127655	BioLegend
TotalSeq™-00098 anti-mouse CD135 Antibody	A2F10	135316	BioLegend
TotalSeq™-00105 anti-mouse CD115 (CSF-1R) Antibody	AFS98	135533	BioLegend

### CITE-seq analysis

Raw sequence data demultiplexing, barcode processing, alignment (mm10) and filtering for true cells were performed using the Cell Ranger Single-Cell Software Suite (v6.0.2), yielding 89,537 cells (WT: 11,326 cells per sample, AML: 11,059 cells per sample) with a mean of 23,788 reads/cell (91.38% mapping rate), median of 1,676 genes/cell, 20,037 total unique detectable genes, and 5,204 median UMI counts/cell. Subsequent filtering for high quality cells, and downstream analyses were performed using Seurat (v4) (30) (**Supplementary Figures 1A, B**). Genes expressed in less than 3 cells and cells that express less than 300 genes were excluded from further analyses. Additional filtering of cells was determined based on the overall distributions of total RNA counts (< 80,000) and the proportion of mitochondrial genes (< 10%) detected to eliminate potential doublets and dying cells, respectively. Additional detection of doublets was performed using Scrublet (31) using a cut-off threshold based on total distribution of doublet scores (doublet score < 0.2). Quantification of mitochondrial and ribosomal gene expression was calculated using the PercentageFeatureSet function, using gene sets compiled from the HUGO Gene Nomenclature Committee database. Cell cycle phase scoring was accomplished against normalized expression via the CellCycleScoring function using mouse genes orthologous to known cell cycle phase marker genes. Ultimately, 74,198 high quality cells (WT: 37,033 cells, AML: 37,165 cells) across samples were included in downstream analyses. Ambient RNA correction was performed using SoupX (32). Normalization and variance stabilization of gene expression data were conducted using regularized negative binomial regression (sctransform) implemented with Seurat. Normalization of ADT abundances was accomplished via the centered log-ratio method. Principal component analysis (PCA) was performed on normalized data and optimal dimensionality of the dataset was decided by examination of the Elbow plot, as the total number of PCs where gain in cumulative variation explained was greater than 0.1% (PCs = 41). Unsupervised cluster determination was performed against a constructed SNN graph via the Louvain approach using a resolution of 0.08. UMAP was applied for non-linear dimensionality reduction to obtain a low dimensional representation visualization of cellular states. Differential expression between clusters or samples was determined using the MAST method (33) via the FindMarkers function, using a minimum expression proportion of 25% and a minimum log fold change of 0.25. Mean expression of markers found within each cluster or cell annotation were used for subsequent analyses including dotplot visualization. Major cell lineages were determined using SingleR (34) using gene sets derived from the ImmGen database. Reference based mapping of the DC compartment was performed against a previously published dataset characterizing DC heterogeneity in mouse spleen [Brown et al. (5)]. Raw counts were obtained from the Gene Expression Omnibus (GSE137710) and re-processed utilizing a Seurat based pipeline described above. Reference based mapping and label transfer of previously annotated cells was performed using the FindTransferAnchors and MapQuery functions implemented within Seurat. DC-specific annotations were further refined using curated marker assessment. Unbiased T-cell phenotype annotation was performed using ProjecTILs (35) and refined using knowledge-based assessment of canonical gene markers.

### Statistics

A 2-sample Student t test with unequal variances was used to test for a statistical difference between 2 groups or a 1-sample Student t test was used to test for a statistical difference in the percent change from 0% where indicated. Unless otherwise indicated, all hypothesis tests were 2-sided, and a significance level of 0.05 was used. Ordinary One-Way ANOVA used to test-for statistical differences between 3 groups. Statistics and Graphs were generated by using Prism 10 software (GraphPad Software https://www.graphpad.com/).

## Results

### Bone marrow samples from patients with AML exhibit disrupted development of cDCs

**Figure 1:**
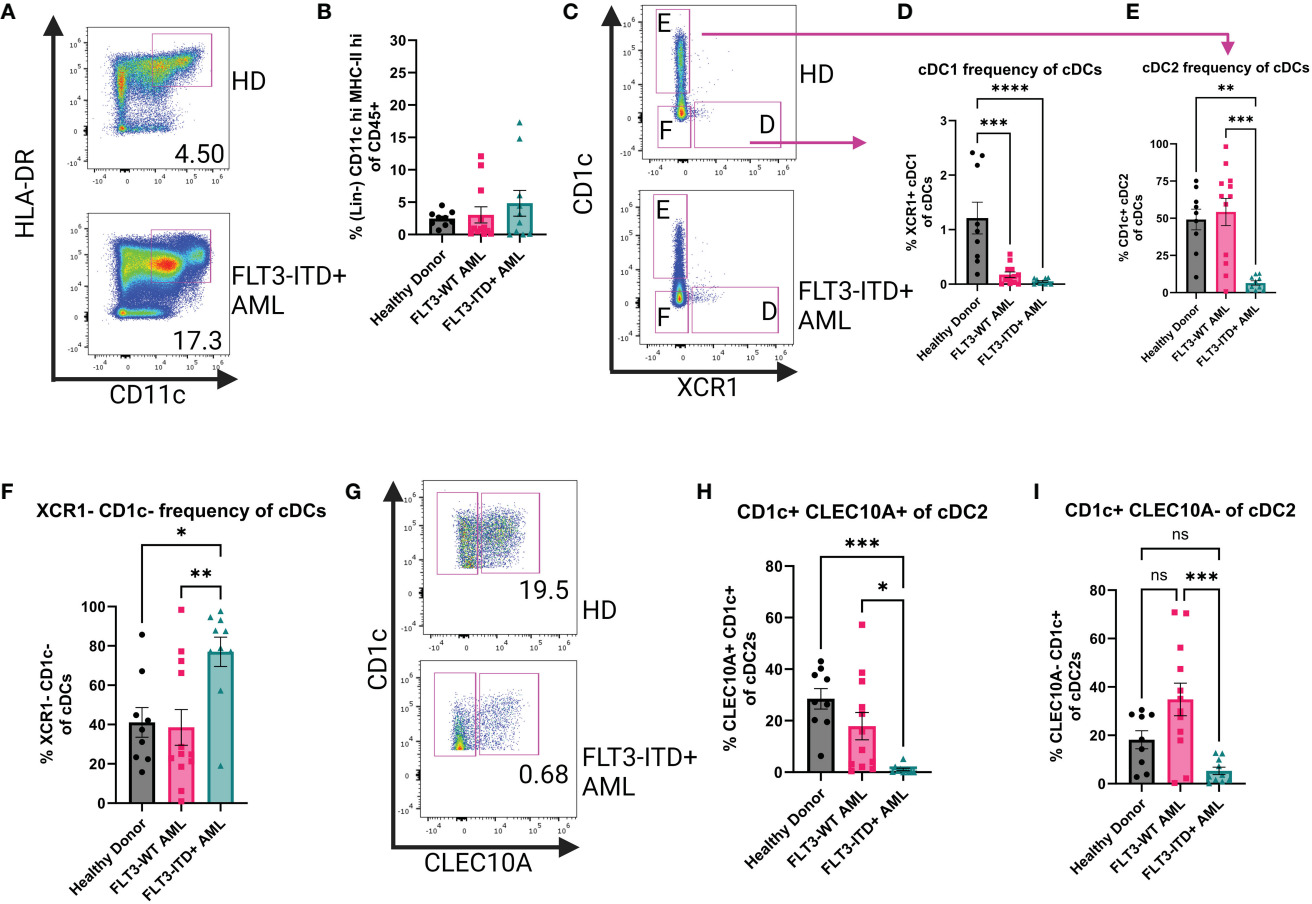
Human Bone Marrow Phenotyping Reveals Disruption of cDC Populations. **(A)** Representative dot plots showing human bone marrow cDCs. cDCs defined as Lin(CD3e, CD19, CD56, CD14, AXL, CD123)-CD45^+^CD11c^+^HLA-DR^+^. **(B)** Summary graph for **(A)** Each symbol is one human sample. HD n=9, FLT3-WT AML n=12, FLT3-ITD^+^ AML n=10. **(C)** Representative dot plots showing cDC1 and cDC2 subsets of the cDCs identified in **(A)**. cDC1 defined as CD1c-XCR1^+^ and cDC2 defined as XCR1-CD1c^+^. **(D–F)** Summary graphs for **(C)** Each symbol is one human sample. HD n=9, FLT3-WT AML n=12, FLT3-ITD+ AML n=10. **(G)** Representative dot plots showing CLEC10A expression of cDC2s. **(H, I)** Summary dot plots of CLEC10a+ and CLEC10a- cDC2s as a frequency of total cDCs **(A)**. * = P < 0.05; ** = P < 0.01; *** = P < 0.001; **** = P < 0.0001; ns = P > 0.05.

Bone marrow aspirates from patients with AML were analyzed by flow cytometry to measure frequencies of cDCs based on their expression of high levels of CD11c and HLA-DR on their surface (**Figure 1A**) after gating out non-DC immune cells (**Supplementary 2A**). We investigated AML Blast gating on our patient samples to identify any significant enrichment of CD11c+ HLA-DR+ cells and did not find that our DC populations are within the Blast compartment (**Supplementary Figure 3**). We found that DCs from patients with AML showed a heterogeneous phenotype compared to healthy donors (HD). For a subset of patients, the frequency of cDCs as a proportion of CD45+ cells were increased compared to HD and this trend was even more pronounced in the samples from FLT3-ITD+ patients (**Figure 1B**). We then sought to investigate if there were any changes to the major cDC subtypes. We therefore assessed the expression of CD1c and XCR1 to identify cDC2 and cDC1, respectively (**Figure 1C**). The frequency of XCR1+ cDC1s was significantly lower in both AML patient groups compared to HD (**Figure 1D**). The frequency of CD1c+ cDC2 was significantly lower in FLT3-ITD+ patient samples but there was no difference between HD and FLT3-WT AML (**Figure 1E**). Unexpectedly the CD1c XCR1 double-negative population was significantly higher in the FLT3-ITD+ AML group compared to HD and FLT3-WT (**Figure 1F**). It has been recently appreciated that cDC2 can be further subtyped by their expression of cell surface CLEC10A as an alternative to intracellular T-bet staining (5) and therefore measured CLEC10A expression on CD1c+ cDC2s (**Figure 1G**). We observed that the frequency of CLEC10A+ CD1c+ cells was significantly lower in the FLT3-ITD+ AML group compared to HD and FLT3-WT AML (**Figure 1H**) and the same was true for the CLEC10A- cDC2s (**Figure 1I**). Our flow cytometry data is similar to what was found from bulk-RNA sequencing analysis of “DC-Like” signatures from the BEAT AML dataset (n=531), where subsets of AML patients with FLT3-ITD had increased “DC-Like” score compared to HD (**Supplementary 2B**) (14, 36). Taken together, this data suggests that in some patients with FLT3-ITD+ AML, the homeostasis of bone marrow cDCs is disrupted and is characterized by a previously unreported expansion of double-negative XCR1-cDC1- poorly-differentiated cDCs.

### Changes in cDCs in FLT3-ITD+ mouse model

**Figure 2:**
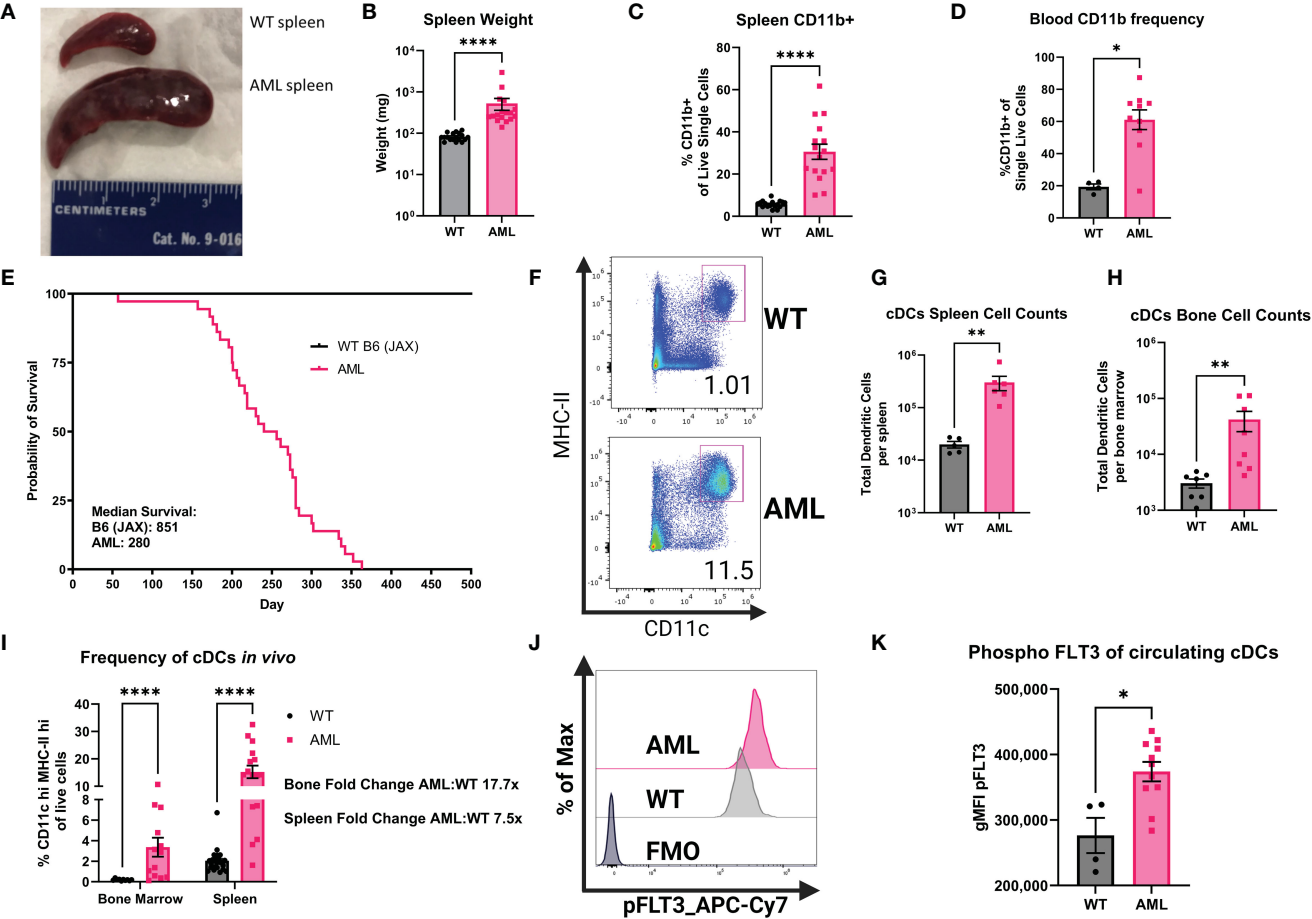
AML mice have increased frequencies of myeloid cells and cDCs. **(A)** Comparison of dissected spleens from healthy WT mice and diseased AML mice. Ruler for scale (cm). **(B)** Summary graph of spleen wet weight for WT and AML mice. Each symbol is one mouse. WT n=18 AML n=17. **(C)** RBC lysed single-cell suspensions of WT and AML mice spleens were stained with monoclonal antibodies for known immune cell markers. Summary graph for frequency of CD19-CD3e-CD11b^+^ splenocytes. Each symbol is one mouse. WT n=17 AML n=16. **(D)** RBC lysed single-cell suspensions of WT and AML PBMCs were stained with monoclonal antibodies for known immune cell markers. Summary graph for frequency of CD19-CD3e-CD11b^+^ PBMCs. Each symbol is one mouse. WT n=4 AML n=10. **(E)** Survival curves of reference C57BL/6J mice from Jackson Laboratories (B6) and our mouse model AML mice. **(F)** Representative dot plots showing mouse splenic cDCs. cDCs defined as Lin(Ly6C, Ly6G, F4/80, CD3e, CD19, NK1.1)- CD45^+^CD11c^hi^MHC-II^hi^. **(G)** Summary graph of cDC cell counts per spleen of WT and AML mice. Each symbol is one mouse. WT n=5 AML n=6. **(H)** Summary graph of cDC cell counts per bone marrow of WT and AML mice. Each symbol is one mouse. WT n=5 AML n=6. **(I)** Summary graph of **(F)**. Each symbol is one mouse. WT bone marrow n=10 WT spleens n=23. AML bone marrow n=13 AML spleens n=16. **(J)** Representative histograms of phospho-FLT3 (pFLT3) staining in cDCs as measured by flow cytometry. RBC lysed single-cell suspensions of WT and AML PBMCs were stained with monoclonal antibodies for known immune cell markers at the surface and were stained for pFLT3 in the cytoplasm. **(K)**. Summary graph showing geometric mean fluorescent intensity (gMFI) pFLT3 staining of blood circulating cDCs. Each symbol is one mouse. WT n=4 AML n=11. * = P < 0.05; ** = P < 0.01; **** = P < 0.0001.

To better understand how the FLT3-ITD mutation in AML affects cDCs, we characterized cDCs in a mouse model of AML. Our lab has previously published work using AML mice that harbor the FLT3-ITD mutation and spontaneously develop AML to interrogate T cell dysfunction (37, 38). Here we utilized mice that express one copy of FLT3-ITD under the endogenous FLT3 promoter, have a homozygous loss of TET2 and a homozygous loss of p53 using Cre-mediated deletion of LoxP-flanked alleles (AML mice) (21, 28, 39, 40). This model is similar to others who reported combining FLT3-ITD with loss of TET2, but in our model the mutations are limited to the myeloid compartment by use of LysM-Cre, thereby retaining a wild-type lymphoid compartment (41). This combination of mutations produces spontaneous AML, as evidenced by splenomegaly (**Figures 2A, B**) and increased CD11b+ cell frequency in both the spleen and the blood (**Figures 2C, D**). Furthermore, AML mice succumb to disease and die prematurely (**Figure 2E**). We then measured populations of DCs using the canonical surface markers CD11c and MHC-II (**Figure 2F**) which are expressed at high levels by cDCs compared to other myeloid subsets. We found that in both compartments, AML mice exhibited significantly increased cDCs (**Figures 2G, H**). When comparing the frequency of cDCs in the bone marrow and spleen to healthy WT controls we found that cDCs were significantly more abundant in AML mice (**Figure 2I**). The phenotype observed in AML mice was not observed in littermates that have wild-type FLT3 but still harbored loss of both TET2 and p53 (**Supplementary Figure 4**), supporting our hypothesis that FLT3-ITD would lead to increased abundance of cDCs *in vivo*. To confirm that FLT3-ITD has constitutive signaling in cDCs, we performed intracellular flow cytometry to measure phosphorylated FLT3 (pFLT3) using an antibody that recognizes Y591 of the intracellular domain of FLT3 (**Figure 2J**) (42, 43). When comparing the geometric mean fluorescent intensity (gMFI) between WT and AML we found that circulating cDCs in AML blood have more pFLT3, as expected in mice with a FLT3-ITD mutation (**Figure 2K**). In agreement with our gating approach used with human patient samples, we evaluated the GEMM tumor phenotype within bone marrow and spleen to which we did not observe an enrichment of DC-phenotype markers (**Supplementary Figure 5**), confirming that our gating criteria are selective for differentiated cDCs in our GEMM data. Taken together, our AML mouse model displays significant expansion of cDCs as a result of FLT3-ITD.

### Single-cell profiling of splenic cDCs in the context of FLT3-ITD AML

**Figure 3:**
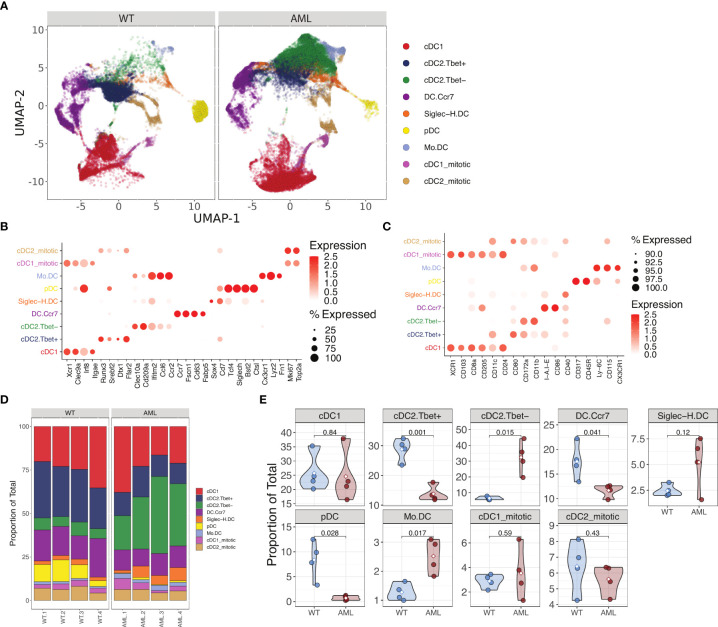
Single cell RNA-seq analysis of AML and WT mouse spleens identify changes in DC populations. **(A)** UMAP plot of DC subsets identified from WT and AML mice after magnetic bead enrichment for DCs. **(B)** Mean expression of various DC gene markers across annotated DC subtypes. **(C)** Mean abundance of protein markers across annotated DC subtypes. **(D)** DC subtype proportions across samples AML (n=4) and WT (n=4). **(E)** Violin plots of DC subtype proportions compared between WT and AML groups. Differences in means were determined using Student’s t-test.

After confirming the phenotype of DCs in our AML mice, we interrogated how DCs are altered in the FLT3-ITD AML environment at the single-cell level using paired transcriptome and cell surface protein profiling (CITE-seq). In total, 74,198 high-quality cells were profiled across samples (WT: n = 4, AML: n = 4) after quality assessment and filtering procedures (**Supplementary Figures 1A, B**). Unsupervised cell clusters were annotated to major cell lineages using a combination of supervised and knowledge-based cell annotation procedures and allowed for the identification of bona fide cDCs from other immune cell subsets as well as any possible AML-tumor related myeloid cells from spleen tissue (**Supplementary Figures 6D, E**). As expected, splenic cells captured from AML mice had a significantly higher proportion of DCs relative to WT mice (**Supplementary Figure 6E**). Cell surface protein abundances measured by Antibody Derived Tags (ADTs) confirmed major cell lineages determined by transcriptome-based annotations (**Supplementary Figures 7A, B**), and strongly correlated with corresponding transcripts across cells (**Supplementary Figure 7C**). Having isolated the DC compartment from other immune cells in the WT and AML splenic environment, we next sought to further define DC phenotype heterogeneity and how it is altered in AML. Recent single-cell profiling of DCs has established further heterogeneity than previously thought, for instance that cDC2 subsets can be segregated by their expression of T-bet (5). To identify DC subtypes within our mouse splenic cells, we implemented a referenced based classification approach utilizing single-cell data derived from mouse splenocytes reported by Brown et al. (5) (**Supplementary Figure 8**) to help in delineating DC phenotypes in our data. Using this approach paired with subsequent marker based assessment, we detected 9 distinct DC subsets including cDC1, T-bet- and Tbet+ cDC2, migratory *Ccr7* expressing DCs (CCR7+ DC), plasmacytoid DCs (pDC) and Siglec-H expressing pDC precursors (Siglec-H DC), monocyte-like DCs, as well as proliferative DC subsets which were strongly supported by canonical marker assessment (**Figures 3A–C**). Notably, upon examination of DC subtype proportions between WT and AML splenocytes, we observed a strong and significant shift in cDC2 subsets with AML splenocyte cDC2s being dominated by a Tbet- phenotype, while WT cDC2s maintained a Tbet+ phenotype (**Figures 3D, E**). The expression of *Clec10a* and *Cd209a* were highly expressed on T-bet- cDC2s (**Figure 3B**) in accordance with data published (5). Surface protein detection by ADT confirmed that T-bet- cDC2s had low expression of CD80 but high expression of CD172a and CD11b as expected (**Figure 3C**). Amongst other DC subtypes, there was no observed difference in the proportion of cDC1s between AML and WT, but significant decreases in pDCs and CCR7+ DCs, and increase in monocyte-like DCs were also observed in AML splenocytes, although to a lesser extent than the shift in Tbet-/+ cDC2s. T-bet- cDC2s were previously reported to preferentially skew naïve CD4^+^ T cells into Th17s *in vitro* (5). Th17 cells have been shown to be elevated in AML patients and have a complicated role in cancer (44, 45). In summary, our data shows that *bona fide* DCs are present in AML and they are skewed towards T-bet- cDC2 phenotype.

### AML mice have disrupted CD4^+^ T cell phenotype and cytokines

**Figure 4:**
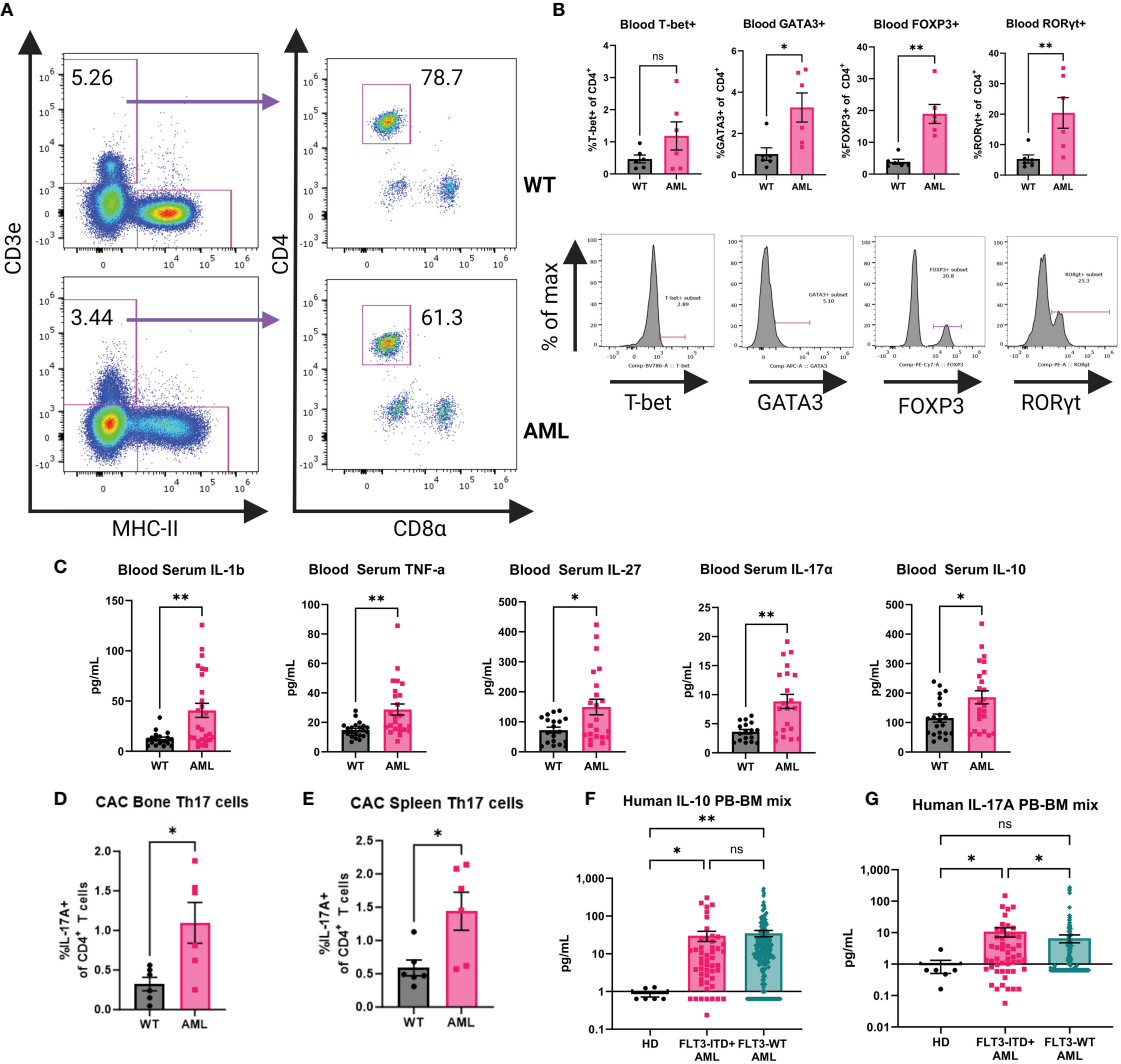
AML mouse blood exhibits a Th17 inflammatory phenotype. **(A)** Representative dot plots for gating on mouse blood CD4^+^ T cells by flow cytometry. Top row is WT and bottom row is AML. **(B)** Transcription factor expression of CD4^+^ T cells. Top row is summary bar charts of transcription factor expression in CD4^+^ T cells from **(A)**. Each symbol is an individual mouse. WT (n=6) and AML (n=6). Bottom row is representative histograms of each transcription factor. **(C)** Blood serum cytokines in WT (n=21) and AML (24) mice. Each symbol is an individual mouse. Summary bar charts for selected inflammatory cytokines measured using BioLegend LegendPlex multiplex assay Mouse Inflammation Panel. **(D)** Summary bar chart for bone marrow IL-17A+ CD4^+^ T cells after stimulation with Cell Activation Cocktail for six hours. WT n=6 and AML n=6. **(E)** Summary bar chart for spleen IL-17A+ CD4^+^ T cells after stimulation with Cell Activation Cocktail for six hours. WT n=6 and AML n=6. **(F)** Summary bar charts for Luminex measurement of human IL-10 cytokine detected in HD and AML patient samples of mixed peripheral blood (PB) and bone marrow (BM). HD (n=6) and FLT3-ITD+ AML (n=50) and FLT3-WT AML (n=251). **(G)** Summary bar charts for Luminex measurement of human IL-17 cytokine detected in HD and AML patient samples of mixed peripheral blood (PB) and bone marrow (BM). HD (n=6) and FLT3-ITD+ AML (n=50) and FLT3-WT AML (n=251). * = P < 0.05; ** = P < 0.01.

It has been shown that mature DCs tune adaptive T cell responses into T-helper subsets (e.g. Th1, Th2, Th17, and Treg) (46–48) and given that the AML mice contain significantly increased levels of cDCs *in vivo* we hypothesized that CD4^+^ T cell populations would be altered systemically in the context of AML. We sampled peripheral blood from AML and WT healthy mice to measure the frequency of CD4^+^ T cells (**Figure 4A**). The frequency of circulating CD4^+^ T cells was not statistically different (**Supplementary Figures 4G, H**) but when we compared the canonical T-helper transcription factors T-bet, GATA3, FOXP3, and RORγt we found that in AML mice they had increased Treg and Th17 populations (**Figure 4B**). Examination of the T-cell compartment identified from single-cell profiling of WT and AML splenocytes supported a strong shift away from naïve CD4^+^ T cells and an expansion of Tregs in AML spleen (**Supplementary Figure 9D**). Th17 cells could not be identified given the relatively low number of total T cells available for analysis (**Supplementary Figure 9D**). We further confirmed this T helper phenotype in the AML mice by analyzing the putative T helper transcription factors in the bone marrow and spleen (**Supplementary Figures 10A, B**). We consistently see that in all three tissues (blood, bone, and spleen) that CD4^+^ T cells are skewed towards Treg and Th17 phenotypes.

The increased abundance of cDCs and the altered T-helper compartment led us to hypothesize that the cytokine profile in AML mice would also be altered. Serum samples from AML and WT healthy mice were analyzed by LegendPlex Mouse Inflammation Panel for inflammation associated cytokines (**Figure 4C**). We found that AML mice had increased levels of IL-1β and TNF-α, consistent with a chronic disease phenotype. We also observed increased levels of IL-27 which is a potent DC-secreted cytokine that can influence T cell responses after activation (49, 50) as well as the Treg and Th17 associated cytokines IL-10 and IL-17A (**Figure 4C**). To confirm the source of IL-17A in our GEMM, we performed *ex vivo* stimulation of bone marrow and splenocytes from WT and AML mice. We stimulated cells with either Cell Activation Cocktail containing PMA+ Ionomycin and Brefeldin A (CAC) or anti-CD3ϵ+anti-CD28 and performed intracellular cytokine staining and flow cytometry analysis. In both bone marrow and spleen tissue we see increased IL-17A in the AML samples when compared to WT mice. We identified CD4^+^ T cells as the source of IL-17A (**Figures 4D, E**, **Supplementary Figure 11A**). Furthermore, we analyzed the non-T cell compartment by analyzing CD3ϵ- cells in our assay and we do not find a detectable presence of IL-17A in non-T cells (**Supplementary Figure 11B**). Based on these findings we analyzed serum from bone marrow and peripheral blood of HD, FLT3-ITD+ AML, and FLT3-WT patient samples by Luminex. We saw significantly increased levels of IL-10 in both AML groups compared to HD, but we do not see a difference between AML groups (**Figure 4F**). There is a significant difference of IL-17A detected in patients in the FLT3-ITD+ AML group when compared to both HD and FLT3-WT AML (**Figure 4G**). Together, these data suggest that in the context of AML, naïve CD4^+^ T cells are directed towards Th17 and Treg phenotypes by cDCs.

### AML mice support expansion of OT-II cells *in vivo* and AML DCs promote Th17 skewing *in vitro*

**Figure 5:**
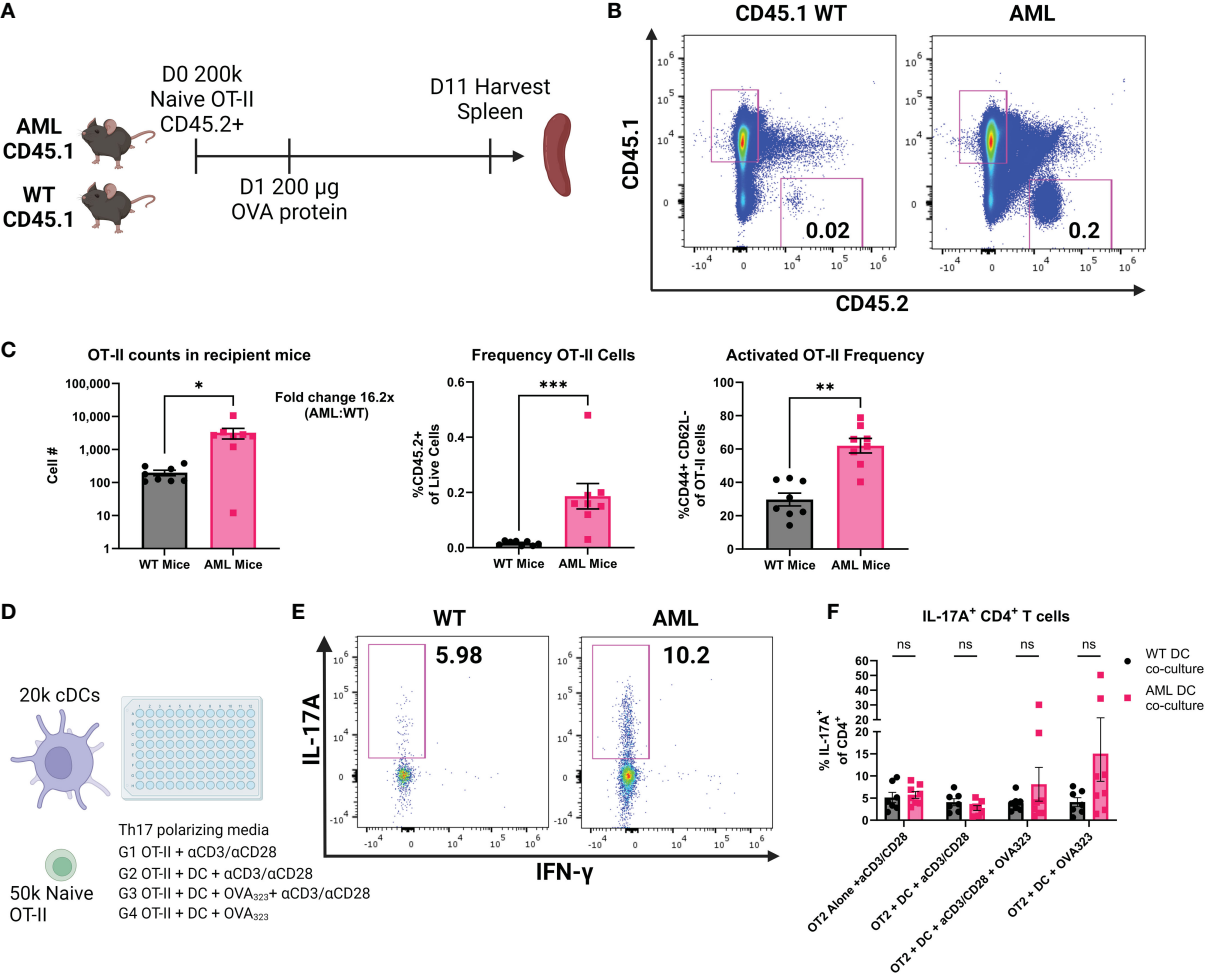
AML mice retain more OT-II cells and AML DCs promote IL-17A production. **(A)** Experimental outline of OT-II adoptive cell transfer. CD45.1+ AML (n=8) and WT (n=8) mice are injected intravenously with 200k naïve CD45.2+ OT-II cells on Day 0. 24 hours later mice were injected intraperitonealy with 200 µg whole OVA protein. 10 days later mice were sacrificed and spleens were harvested for flow cytometry analysis of remaining CD45.2+ OT-II cells. **(B)** Representative dot plots of spleen resident CD45.2+ OT-II cells by flow cytometry. Frequency of live ingle cells noted in the plots. **(C)** Summary bar charts of OT-II cell counts (left panel) and frequency of live single cells (right panel). Each symbol is an individual mouse and summary data collected from two experiments. AML (n=8) and WT (n=8). **(D)** Experimental outline of *in vitro* co-cultures of OT-II cells and cDCs. Naïve OT-II cells were magnetically sorted from spleen tissue using BioLegend MojoSort Mouse CD4 Naïve T Cell Isolation Kit. After magnetic sorting samples were verified for purity by flow cytometry. cDCs were magnetically isolated using BioLegend MojoSort Mouse CD11c+ beads. After magnetic sorting samples were verified by flow cytometry. OT-II cells were plated at 50k cells per well and cDCs were plated at 20k cells per well for five days in Th17 polarizing media using CellXVivo Mouse Th17 Cell Differentiation Kit CDK017 from R&D Systems. **(E)** Representative dot plots of IL-17A+ OT-II cells by flow cytometry. Frequency of CD4^+^ cells noted in the plots. **(F)** Summary bar charts of IL-17A+ CD4^+^ T cells from **(E)**. Each symbol is an individual sample and summary data collected from four experiments. AML (n=8) and WT (n=7). * = P < 0.05; ** = P < 0.01; *** = P < 0.001.

Given that we found the increase in T-bet- cDC2s and Th17 cells in AML mice, there was reason to investigate whether the CD4^+^ T cell phenotype was the result of interactions with DCs. We performed adoptive cell transfer (ACT) using naïve transgenic OT-II cells that are specific to OVA peptides (29, 51, 52) to study antigen specific activation of CD4^+^ T cells. Age-matched healthy WT or AML mice received equal numbers of naïve OT-II cells and one day following received an equal dose of whole-OVA protein to evaluate DC-specific priming of transferred OT-II cells. Spleens were harvested for flow cytometric analysis 10 days after OVA injection (**Figure 5A**). Transferred cells were identified using congenic markers (**Figure 5B**). When probed for transcription factors FOXP3 and RORγt we did not see expression of either transcription factor. We did observe significantly more CD44+ OT-II cells and with increased frequency in AML host mice at Day 11 suggesting that AML are more supportive of CD4^+^ T cells after transfer (**Figure 5C**), suggesting DC-induced priming of naïve OT-II cells. To confirm our findings we also analyzed a cohort of mice that were injected with irrelevant antigen whole-BSA protein after adoptive transfer, where we do not observe an activation phenotype (**Supplementary Figure 12**). Since we did not see evidence of specific Th skewing (*e.g.* FOXP3 or RORγt) *in vivo* after adoptive transfer we next tested an *in vitro* approach where isolated cDCs and naïve OT-II T cells were co-cultured with OVA_323_ peptide (**Figure 5D**). After five days of culture we analyzed secretion of IL-17A by flow cytometry (**Figure 5E**). In the groups that had the addition of the OT-II dominant antigen OVA_323,_ we saw an increased trend of IL-17A secretion in the AML cDC co-cultures (**Figure 5F**). Taken together our data suggests that AML mice have a DC phenotype that supports CD4^+^ T cell retention and polarization of naïve CD4^+^ T cells into a Th17 phenotype. In summary, we find that in the context of our AML mouse model, DCs inherit the FLT3-ITD mutation and DC homeostasis is disrupted. We also report that CD4^+^ T cells are skewed towards Treg and Th17 phenotypes *in vivo* and adoptively transferred OT-II cells are retained in higher numbers in AML recipients. *In vitro* co-cultures of OT-II cells and DCs resulted in more IL-17A secretion in the AML samples. All of these data together suggest that the increased DC phenotype observed also directly impacts the CD4^+^ T cell compartment, which results in a microenvironment that is tumor supportive.

## Discussion

FLT3-ITD is one of the most common mutations found in AML patients and research focus has been placed mainly on the AML blasts. DCs are the most responsive cell type to FLT3 signaling and they rely on this pathway for development (53, 54). We found that in bone marrow from FLT3-ITD+ AML patients, the frequency of cDCs was heterogeneous and patients could be stratified into high/medium/low frequencies. FLT3-ITD- bone marrow samples also had elevated levels of cDCs in the bone marrow compared to HD, but the highest observed frequencies were in the FLT3-ITD+ AML, suggesting that there are cDC precursors that are not tumor blasts that retain this mutation and expand. Our data shows that in AML the cDC1 and cDC2 subsets are disrupted and the frequency of XCR1/cDC1 double-negative cDCs was significantly higher in FLT3-ITD+ samples. This may suggest that patients with FLT3-ITD+ AML may retain more precursors than fully committed DC cells. Reports on bone marrow frequency of cDCs in the literature are sparse (55) but what we observe is consistent with the data reported, where a subset of AML patients have increased levels of cDCs in their bone marrow. However, that study did not have FLT3-ITD mutation status for patients. The majority of AML-DC reports focus on measuring circulating pDCs and cDCs (23, 56, 57)support the idea that FLT3-ITD+ AML patients have increased DC frequency in the peripheral blood. Immunophenotyping more FLT3-ITD+ AML patients will be important for understanding the changes to DC subsets in the bone marrow and the periphery.

We found that our spontaneous AML mice had a significant DC phenotype. It has been reported previously in a non-AML context that the FLT3-ITD mutation led to cDC expansion systemically (26). We see that in the context of AML the expansion of cDCs is even more profound and was a result of FLT3-ITD *in vivo*. This suggests that in our model, the FLT3-ITD mutation drives a proportion of DC-precursors into mature DCs rather than malignant cells. The molecular understanding of DCs has increased significantly due to advances in sequencing technologies (58–60). We used the models from Brown et al. to inform our scRNA-seq analysis (5). To the best of our knowledge, our report is the first to provide scRNA-seq data on DCs in the context of a model of FLT3-ITD+ AML. In combination with our ADT data, we can confidently identify *bona fide* DCs in the spleen. This has allowed us to more accurately describe changes to the DC population without contamination from other myeloid subsets and tumor blasts that have a shared lineage and could confound our DC findings. Our AML mice had more T-bet^-^ cDC2s, a population of cells that have been shown to promote polarization of naïve CD4^+^ T cells into Th17 phenotypes (5). This finding was unexpected to us because reports suggest that strong FLT3 signaling *in vivo* drives all DC subsets to expand (9, 26, 61). This T-bet^-^ cDC2 cell type is novel and will be the subject of future studies to understand how it is functionally different from other DCs in AML. Our data is concordant with what was reported by Brown et. al., which could have clinical implications for AML patients. The role of Th17 cells in cancer is controversial but in AML they have been found to be deleterious for patient survival (44, 45, 62, 63). Our mouse model of AML recapitulates findings from humans where the inflammatory cytokine milieu is permissive to Th17 polarization (37, 38, 64–67).

We recognize that the DC phenotype we describe in our human samples is different from the mouse DC phenotype. This was not unexpected as AML is a very genetically complicated cancer (14, 15). Our mouse model has a select number of mutations (FLT3-ITD, TET2 KO, and TP53KO) that leads to AML-like disease but is a significantly smaller list of mutations compared to human AML patients (14). It is likely that the increased complexity of mutations in the AML patient samples is impacting DC subsets in a way that we cannot replicate in the GEMM that we use. What may address this discrepancy is the use of ATACseq and scRNA-seq on both mouse and human AML DCs to compare their chromatin accessibility and transcriptomes. Such data would shed light on what differences and parallels exist between species regarding the active sites of transcription and gene expression as a result of inherited leukemia mutations.

It is important in our view to consider the contribution of DCs on the T cell compartment as DCs are continuously supplied to the periphery and difficult to ablate compared to lymphocytes. In summary, we have shown that in some subsets of FLT3-ITD+ AML patients the frequency of bone marrow DCs is increased. Using a mouse model of AML we characterized this increase in DC abundance as a result of FLT3-ITD and that they are skewed towards a T-bet^-^ cDC2 phenotype. These cells are adept at polarizing naïve CD4^+^ T cells into the Th17 subset. This work provides novel scRNA-seq data on DCs and further supports rationale for the need to improve the characterization and function of non-tumor myeloid cells and their impact on anti-tumor responses. This is particularly important with the growing interest in developing immunotherapies for AML (37, 38, 66, 67).

## Data availability statement

The datasets presented in this study can be found in online repositories. The names of the repository/repositories and accession number(s) can be found below: NCBI GEO accession GSE238156.

## Ethics statement

The studies involving humans were approved by Oregon Health and Sciences University IRB. The studies were conducted in accordance with the local legislation and institutional requirements. The participants provided their written informed consent to participate in this study. The animal study was approved by Oregon Health and Sciences University IACUC. The study was conducted in accordance with the local legislation and institutional requirements. Studies involving patients were collected under OHSU IRB 4422 Marc Loriaux, PI. in accordance with all local and institutional requirements. Animal studies were approved and conducted under OHSU IACUC protocol “Immune-based therapeutic approaches for acute myeloid leukemia” IP00000907 Evan Lind, PI.

## Author contributions

PF: Conceptualization, Formal analysis, Investigation, Methodology, Writing – original draft, Writing – review & editing. ML: Conceptualization, Data curation, Formal analysis, Methodology, Writing – original draft, Writing – review & editing. YK: Formal analysis, Methodology, Supervision, Writing – review & editing. JM: Methodology, Writing – review & editing. JC: Methodology, Writing – review & editing. AA: Data curation, Formal analysis, Methodology, Writing – review & editing. EL: Conceptualization, Formal analysis, Funding acquisition, Project administration, Resources, Supervision, Writing – original draft, Writing – review & editing, Data curation, Methodology.
