# Supplementary material for: Leukemic mutation FLT3-ITD is retained in dendritic cells and disrupts their homeostasis leading to expanded Th17 frequency
Source: Front Immunol. 2024 Mar 1;15:1297338. doi: 10.3389/fimmu.2024.1297338 (PMC10943691; doi:10.3389/fimmu.2024.1297338)
Supplement: Supplementary file 1 [file DataSheet_1.docx]

**Supplemental Data Flynn et. al.**


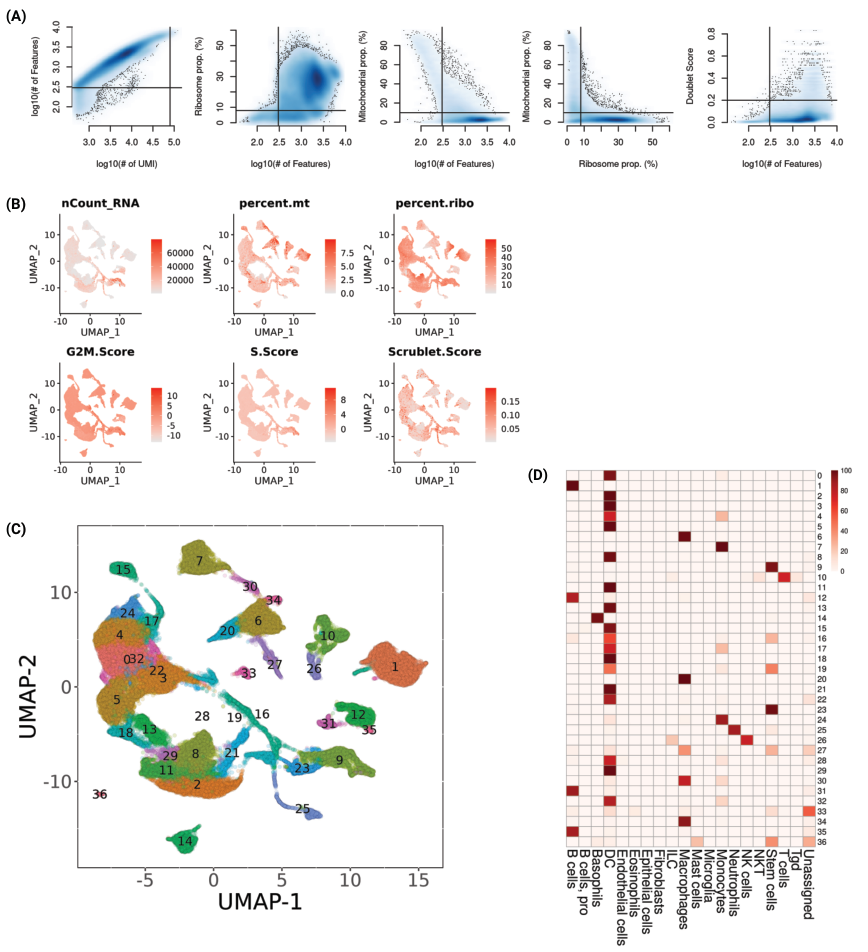


Supplemental Figure 1. Mouse scRNA-seq QC and processing metrics

1. Filtering criteria used to isolate high-quality cells for downstream analysis, including number of detectible features/UMI, ribosomal and mitochondrial gene proportions, and Doublet Score.
2. QC metrics and cell-cycle scoring shown for all cells.
3. UMAP representation of all high-quality cells. Colors represent final clusters determined by unsupervised clustering analysis.
4. Proportion of each cluster annotating to major lineages determined by SingleR supervised classification against the Immgen Database.


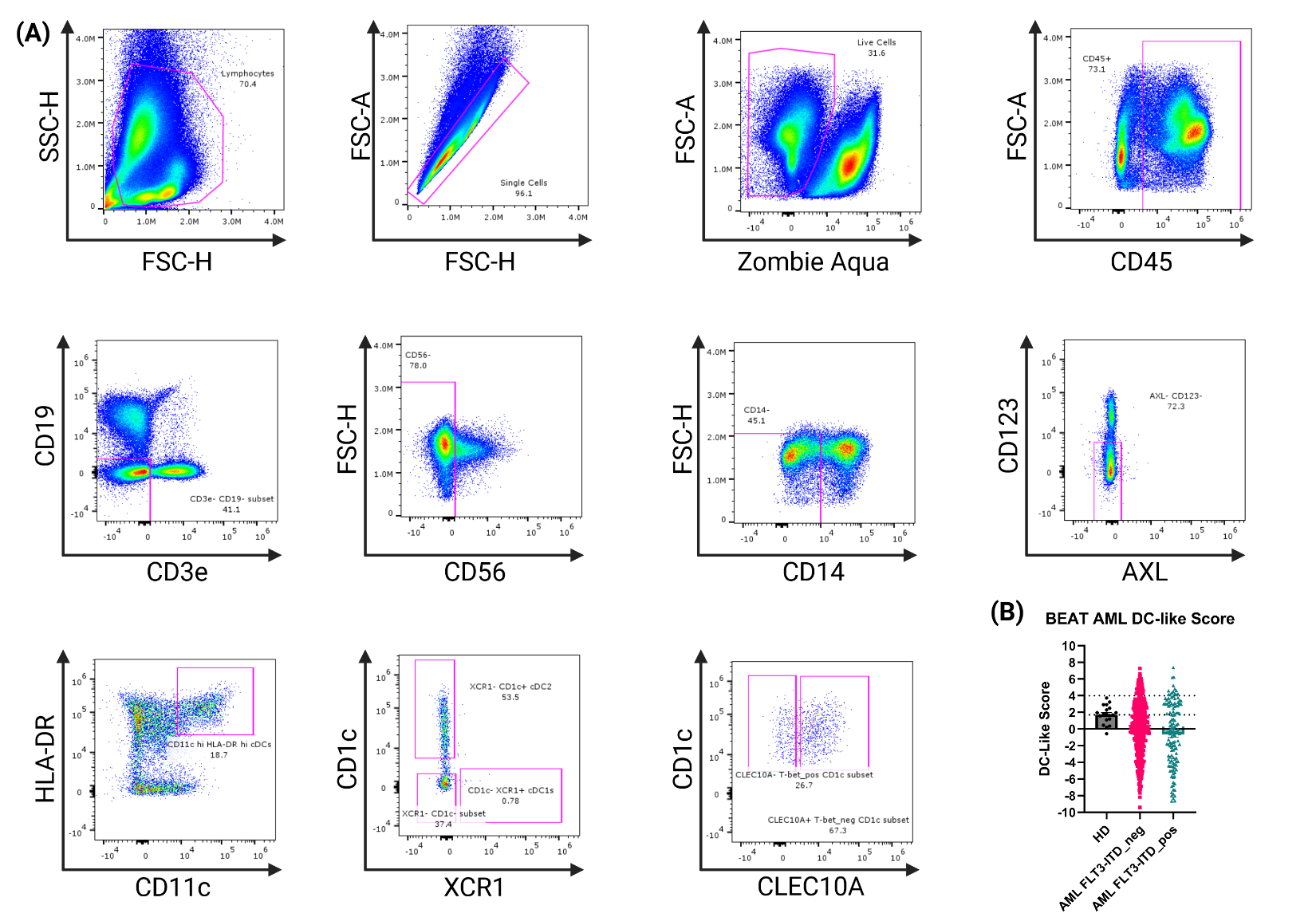


Supplemental Figure 2. Gating scheme for human bone marrow flow cytometry and DC-like score

1. FlowJo gating scheme showing identification of human cDCs from bone marrow aspirate.
2. Dot plots showing DC Cell-Type scores from AML patient samples that were identified using Weighted Gene Co-expression Network Analysis (WGCNA) from BEAT AML Bottomly et. al. 2022 https://doi.org/10.1016/j.ccell.2022.07.002.


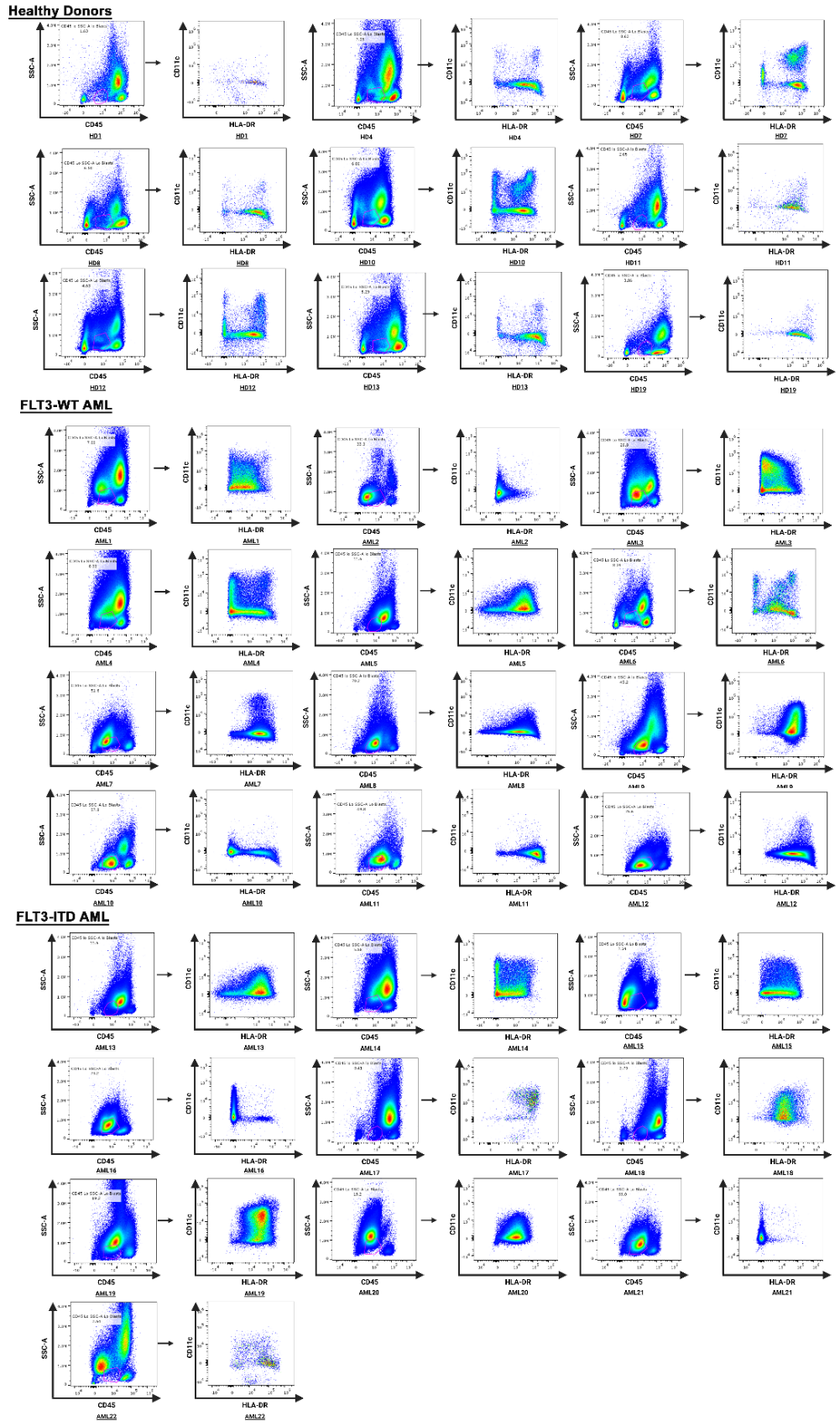


Supplemental Figure 3. AML Blast expression of CD11c and HLA-DR surface proteins

FlowJo dot plot layouts for all analyzed bone marrow aspirate samples (HD n=9; FLT3-WT AML n=12; FLT3-ITD AML n=10). AML Blasts defined as SSC-A low CD45 low.


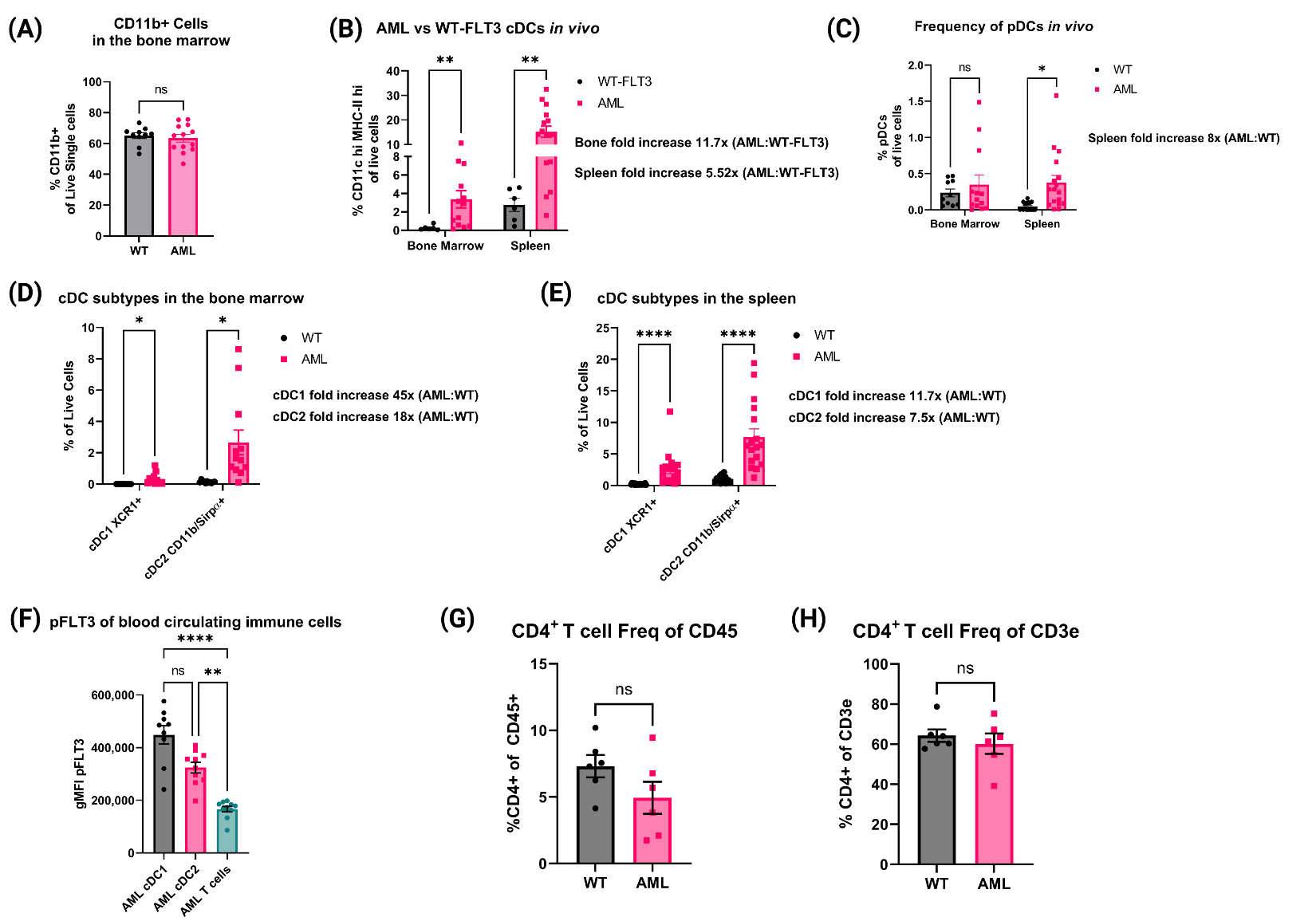


Supplemental Figure 4. Mouse Model of AML Has Significantly Increased cDCs In Vivo

1. Summary bar chart of CD11b+ cell frequency in the bone marrow of WT (n=10) and AML (n=13) mice.
2. Summary bar chart of cDC frequency in the bone marrow and spleen compartments of WT-FLT3 LysM-Cre+ TET2loxp/loxp mice (n=6) and AML mice (n= bone marrow n=13 AML spleens n=16).
3. Summary bar chart of pDC frequency in bone marrow and spleen. Each symbol is one mouse. WT bone marrow n=10 WT spleens n=23. AML bone marrow n=13 AML spleens n=16.
4. Summary bar chart of cDC1 and cDC2 frequency in bone marrow. Each symbol is one mouse. WT bone marrow n=10. AML bone marrow n=13.
5. Summary bar chart of cDC1 and cDC2 frequency in spleens. Each symbol is one mouse. WT bone marrow n=23. AML bone marrow n=16
6. Summary bar chart of blood circulating AML cDC1 and AML cDC2 and AML T cells gMFI pFLT3. N=10.
7. Summary bar chart of blood circulating CD4^+^ T cells as a proportion of CD45+ cells. WT n=6 and AML n=6.
8. Summary bar chart of blood circulating CD4+ cells as a proportion of total CD3ε^+^ T cells. WT n=6 and AML n=6.

 
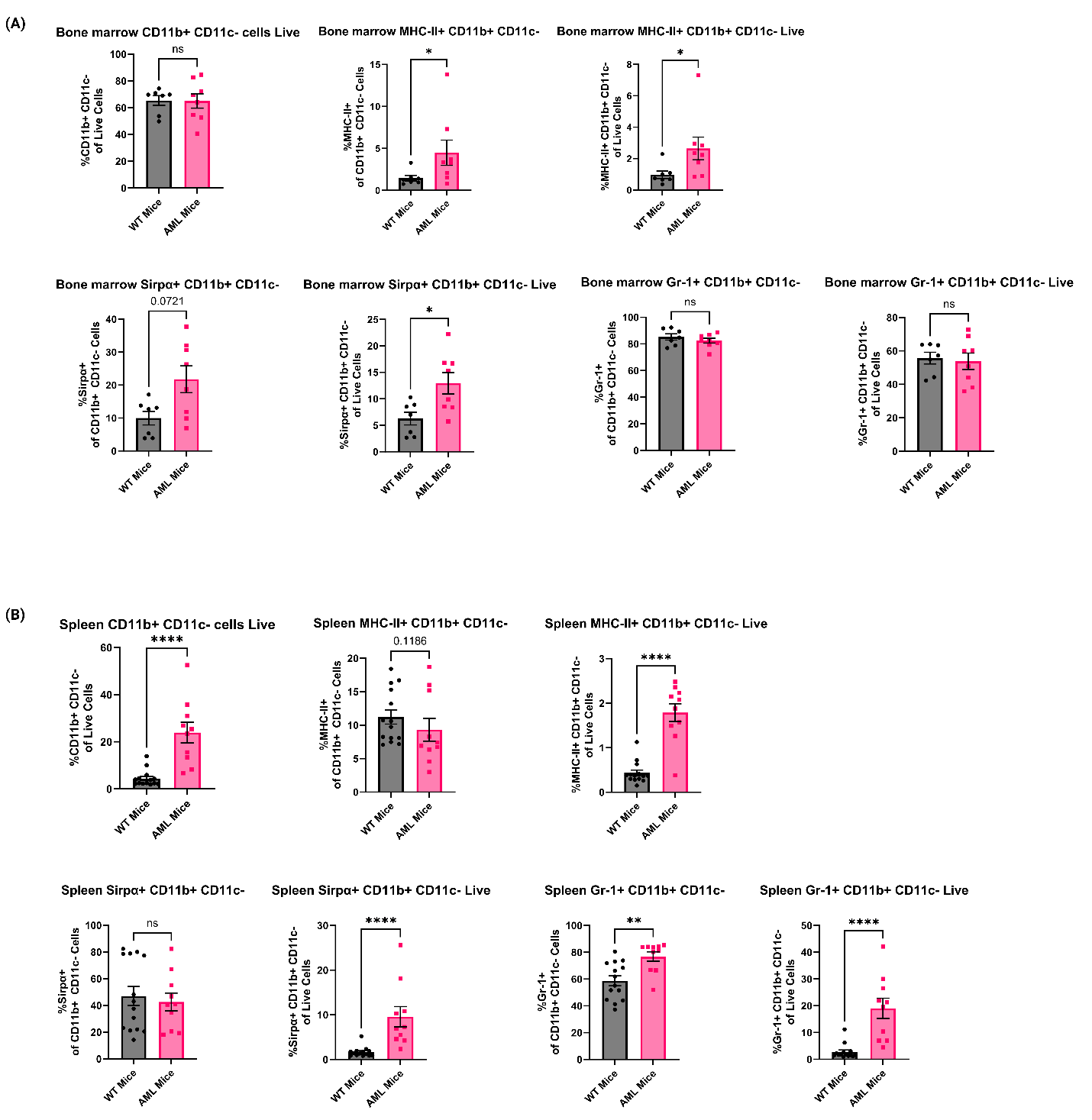


Supplemental Figure 5. GEMM model tumor phenotype in the bone marrow and spleen

1. Summary bar charts of myeloid phenotyping GEMM tumor cells (CD11b+ CD11c-) frequency in the bone marrow of WT (n=7) and AML (n=8) mice.
2. Summary bar charts of of myeloid phenotyping GEMM tumor cells (CD11b+ CD11c-) frequency in the spleen of WT (n=14) and AML (n=10).


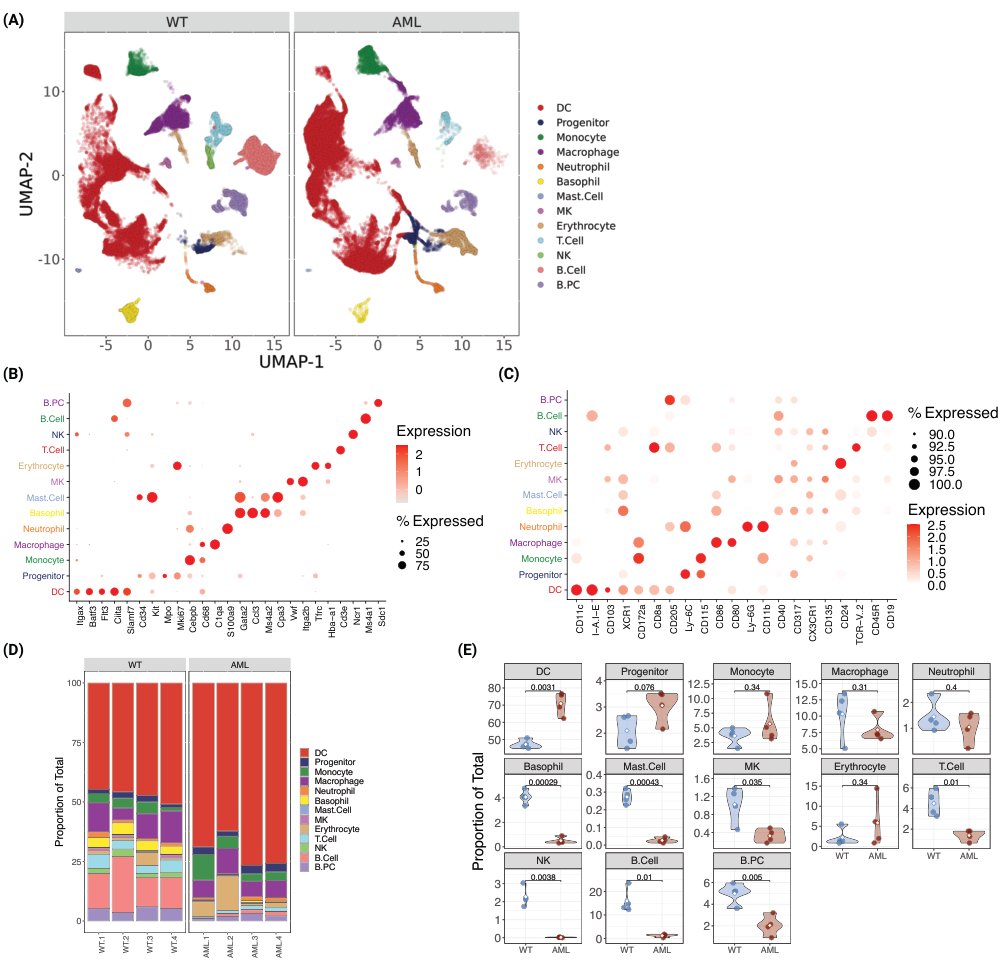


Supplemental Figure 6: Single cell RNA-seq profiling of AML and WT mouse spleens.

1. UMAP plot of total cells derived from WT and AML mice after magnetic bead enrichment for DCs.
2. Mean expression of various linage markers across annotated cell types.
3. Mean abundance of protein markers across annotated cell types.
4. Cell type proportions across samples AML (n=4) and WT (n=4).
5. Violin plots of cell type proportions compared between WT and AML groups. Differences in means were determined using Student’s t-test.


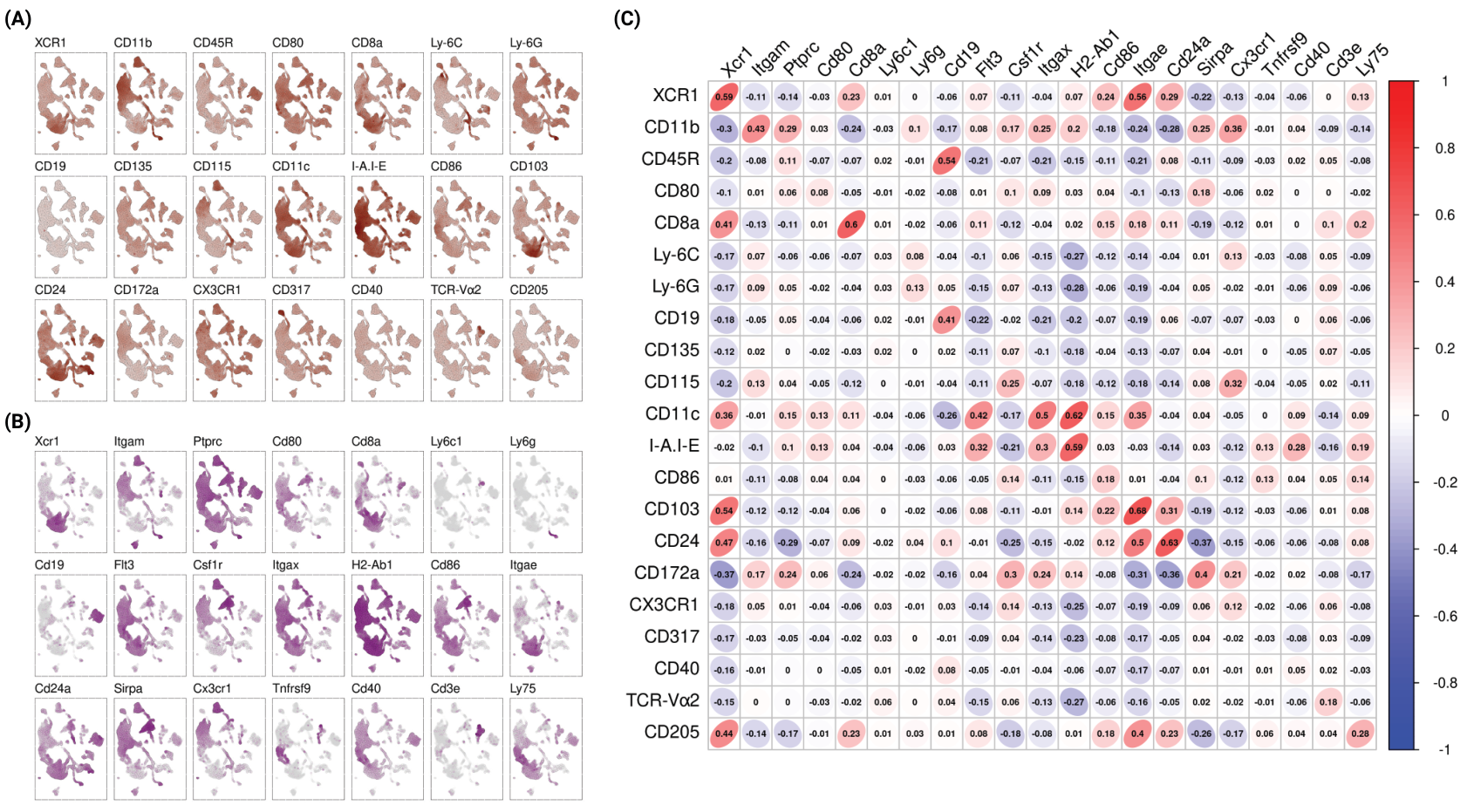


Supplemental Figure 7. Comparison of GEX and Protein ADT profiles across single-cells

1. UMAP representation of for protein ADT abundances.
2. UMAP representation of gene expression markers associated with proteins profiled in A.
3. Correlation matrix for surface proteins and corresponding transcripts across all cells.


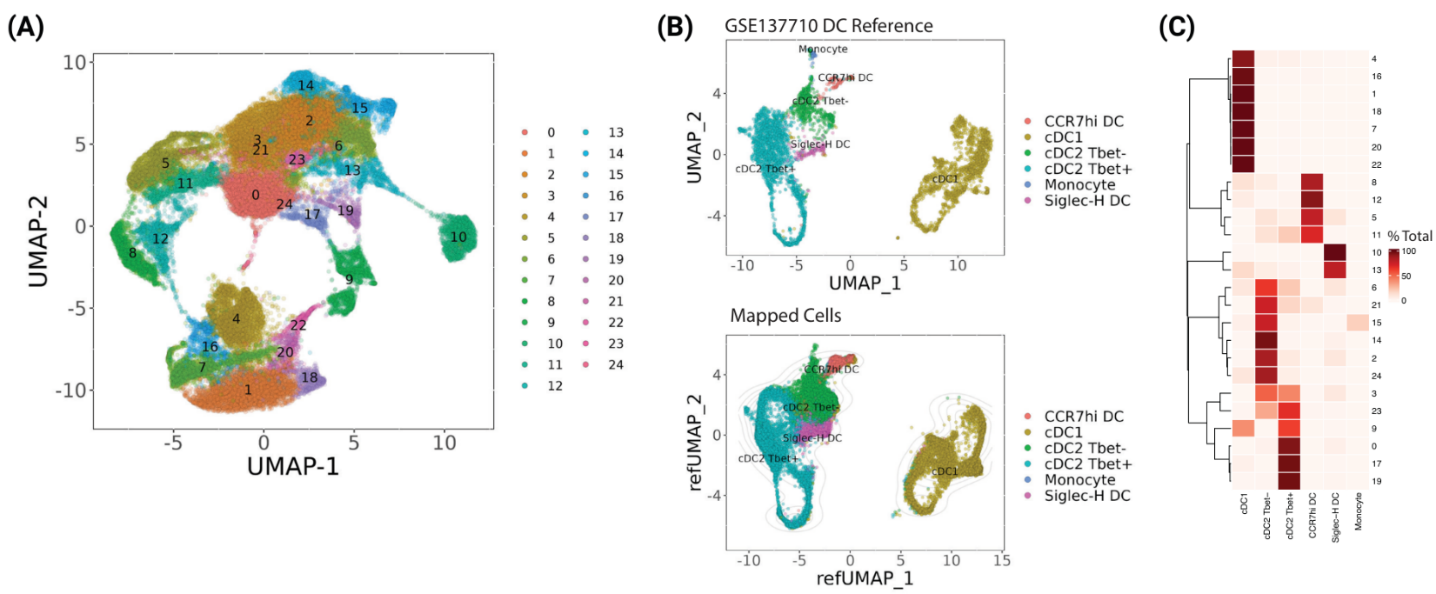


Supplemental Figure 8. Reference based mapping delineates DC heterogeneity in WT and AML mouse spleens.

1. UMAP visualization of the identified DC compartment. Colors represent final clusters determined by unsupervized clustering analysis.
2. Upper panel, GSE137710 DC Reference UMAP from re-analysis of data derived from Brown *et. al.* (GSE137710). Bottom panel, AML and WT mouse splenocytes mapped to Brown *et. al.* reference.
3. Proportion of cells within each cluster annotating to various DC subtypes after classification from reference based mapping.


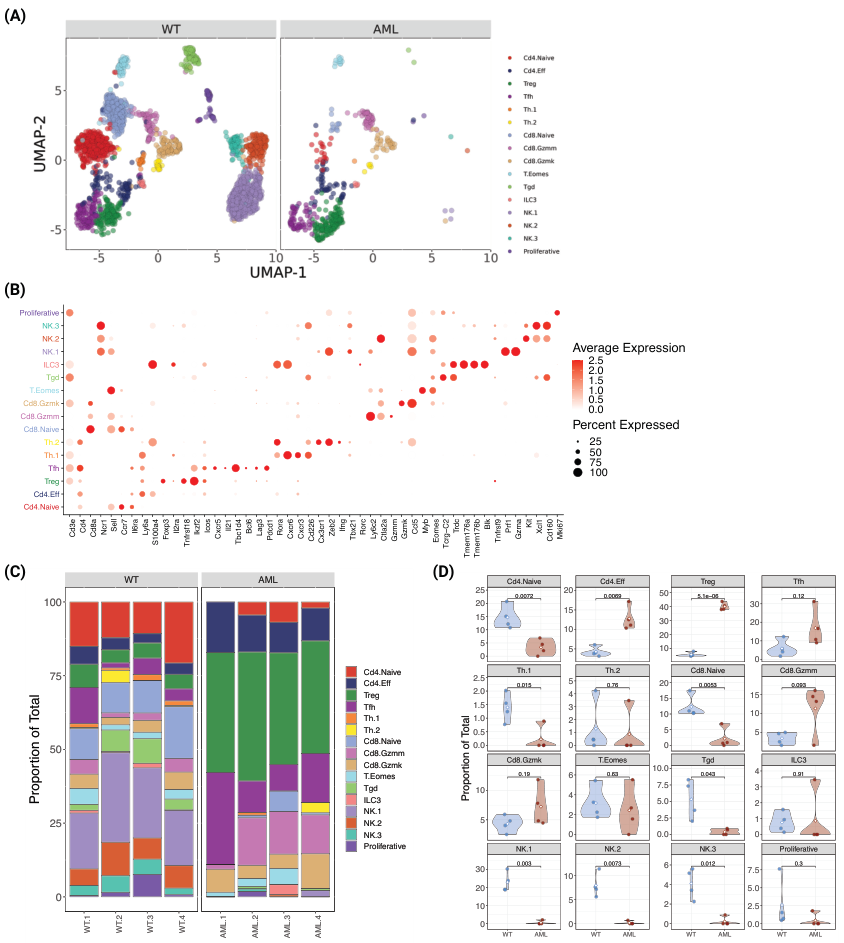


Supplemental Figure 9: Single cell RNA-seq analysis of AML and WT mouse spleens identify changes in T-cell phenotypes.

1. UMAP plot of T-cell and NK subsets identified from WT and AML mice.
2. Mean expression of various T and NK cell gene markers across annotated T-cell subtypes.
3. T and NK cell subtype proportions across samples AML (n=4) and WT (n=4).
4. Violin plots of T and NK cell subtype proportions compared between WT and AML groups. Differences in means were determined using Student’s t-test.


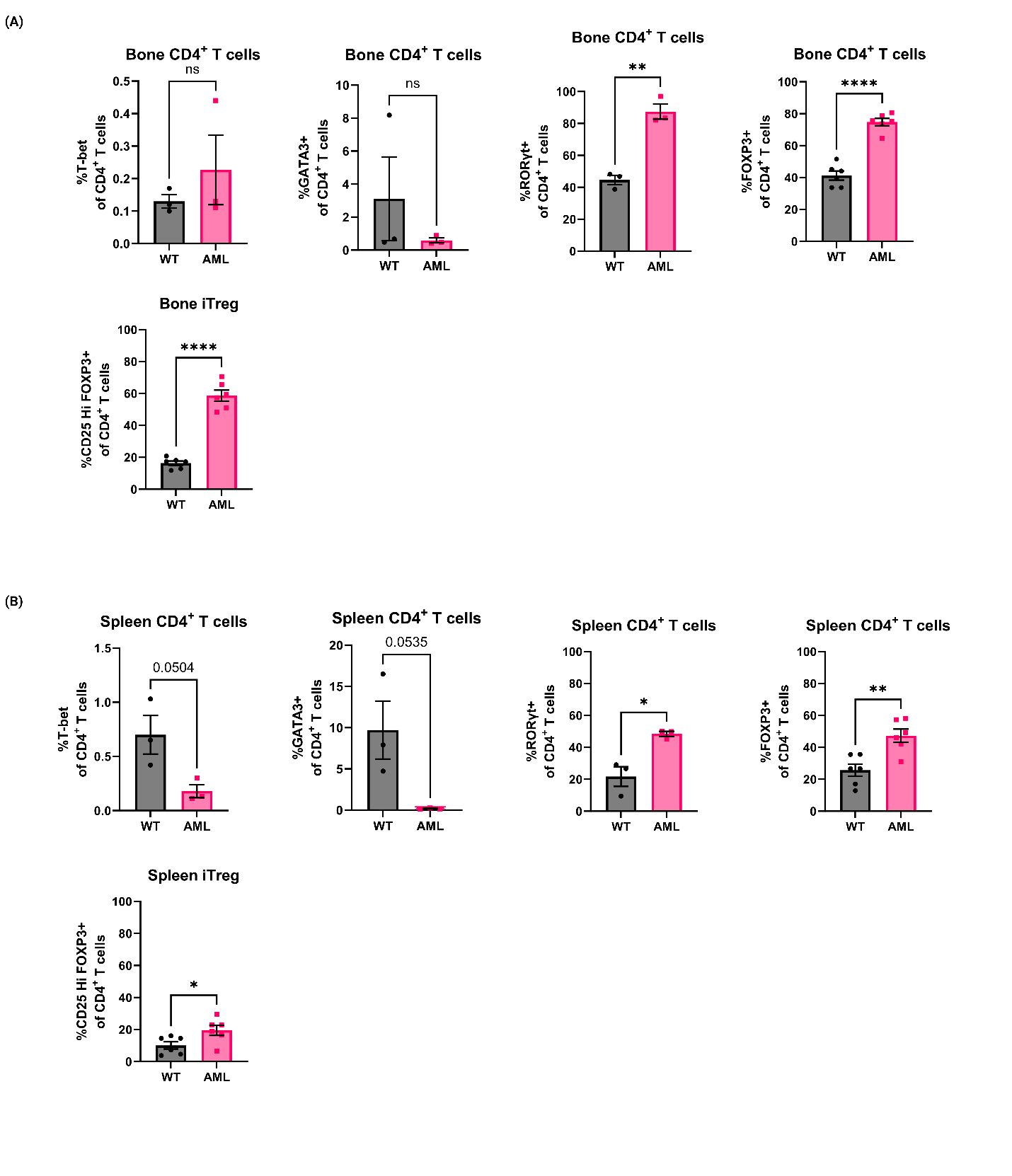


Supplemental Figure 10. GEMM CD4^+^ T cell transcription factor phenotyping in the bone marrow and spleen

1. Summary bar charts of phenotyping GEMM Thelper transcription factors in the bone marrow of WT (n=3) and AML (n=3) mice. FOXP3+ and CD25+ T cell n=6 for both genotypes.
2. Summary bar charts of phenotyping GEMM Thelper transcription factors in the spleen of WT (n=3) and AML (n=3). FOXP3+ and CD25+ T cell n=6 for both genotypes.


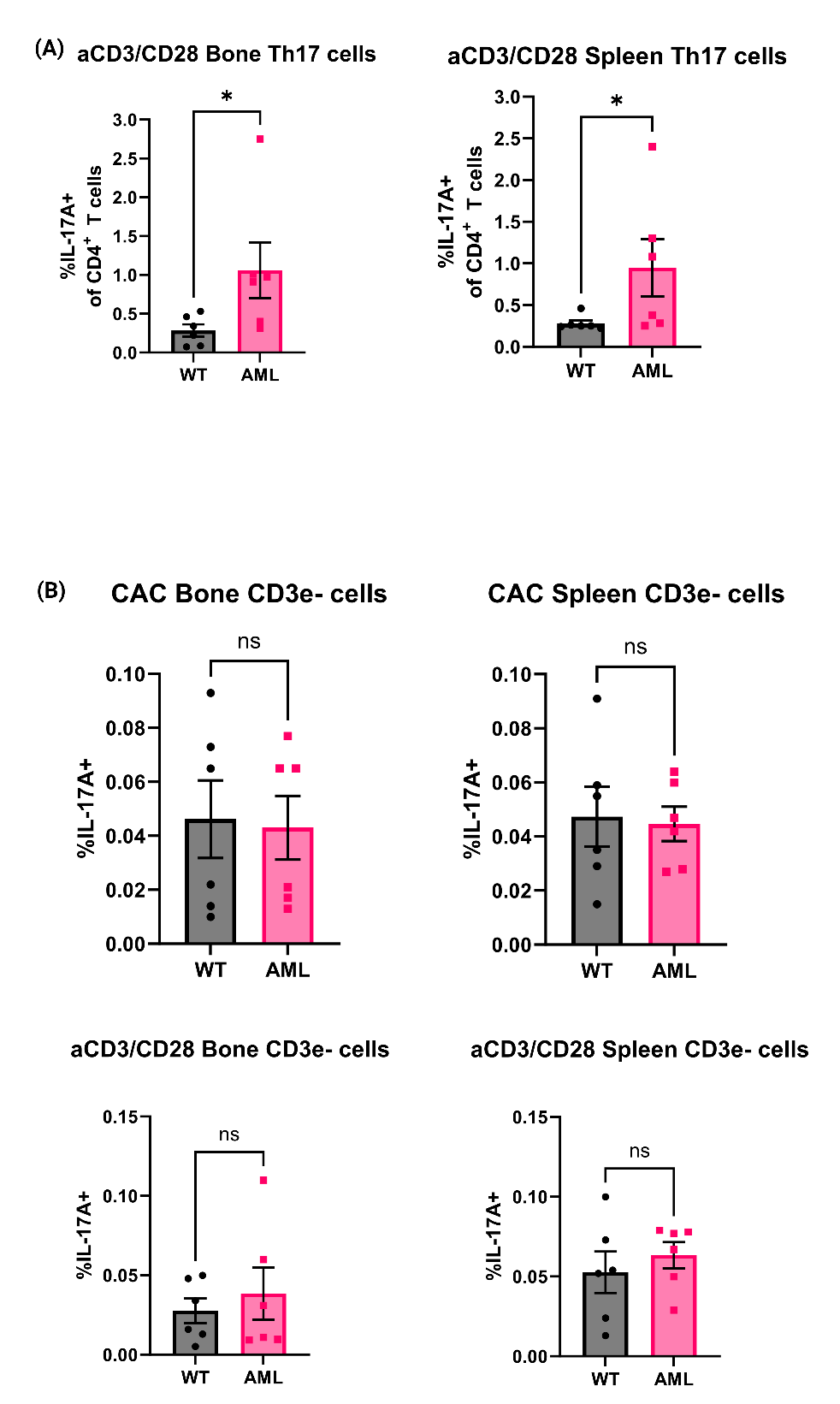


Supplemental Figure 11. IL-17A cytokine detection after *ex* *vivo* stimulation

1. Summary bar charts of IL-17A+ CD4^+^ T cells from the bone marrow or spleens of WT (n=6) and AML (n=6) mice after stimulation with anti-CD3ε anti-CD28 *in vitro* for six hours.
2. Summary bar charts of IL-17A+ events in the CD3ε- compartment of the bone marrow or spleen of WT (n=6) and AML (n=6) mice after stimulation with Cell Activation Cocktail or anti-CD3ε anti-CD28 *in vitro* for six hours.


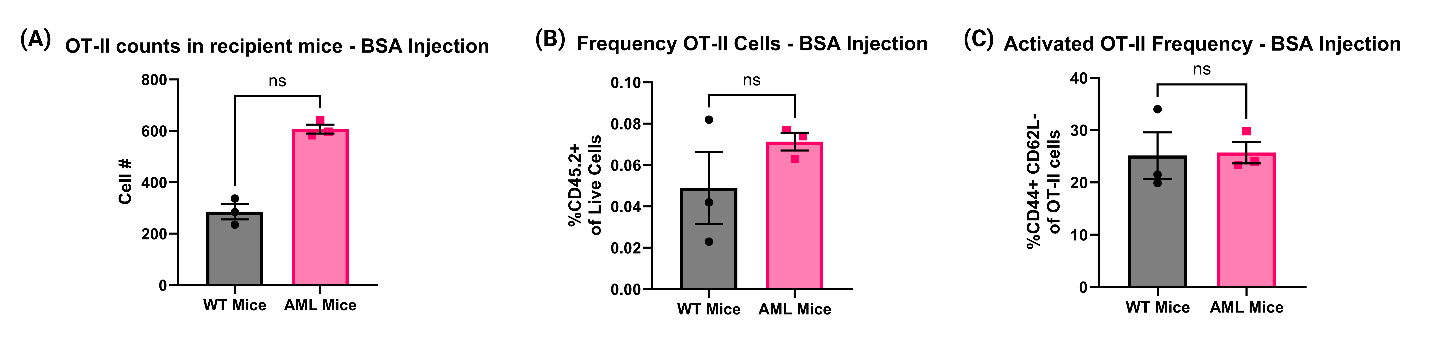


Supplemental Figure 12. OT-II cells are antigen specific and do not respond to irrelevant BSA protein *in vivo*

1. Summary bar chart detectable CD45.2^+^ OT-II cells in the spleens of WT (n=3) and AML (n=3) mice after 11 days post-adoptive transfer into CD45.1^+^ recipients after receiving whole-BSA protein.
2. Summary bar chart frequency of CD45.2+ OT-II cells in the spleens of WT (n=3) and AML (n=3) mice after 11 days post-adoptive transfer into CD45.1+ recipients after receiving whole-BSA protein.
3. Summary bar chart frequency of CD44+, CD62L- OT-II cells in the spleens of WT (n=3) and AML (n=3) mice after 11 days post-adoptive transfer into CD45.1+ recipients after receiving whole-BSA protein.
